# A Reassessment of Phylogenetic Relationships in Class Oligohymenophorea (Protista, Ciliophora) Based on Updated Multigene Data

**DOI:** 10.1002/ece3.70950

**Published:** 2025-02-24

**Authors:** Bailin Li, Yumeng Song, Xiang Wang, Qiyue Zhao, Menghan Liu, Lihui Liu, Xuming Pan, Zhenzhen Yi

**Affiliations:** ^1^ Key Laboratory of Biodiversity of Aquatic Organisms Harbin Normal University Harbin China; ^2^ Guangzhou Key Laboratory of Subtropical Biodiversity and Biomonitoring, School of Life Science South China Normal University Guangzhou China

**Keywords:** ciliates, Loxocephalida, multigene, Oligohymenophorea, phylogeny, Urocentria

## Abstract

Within the ciliate class Oligohymenophorea, many evolutionary relationships among taxa remain unresolved. This study incorporates 97 new sequences from 30 oligohymenophorean populations, including nuclear small subunit ribosomal (SSU‐rRNA) genes, nuclear ITS1‐5.8S‐ITS2 rRNA regions, nuclear large subunit ribosomal (LSU‐rRNA) genes, mitochondrial cytochrome oxidase c subunit I (cox 1) genes, and mitochondrial small subunit ribosomal RNA (mtSSU‐rRNA) genes. With the addition of these new sequences, we performed comprehensive multigene phylogenetic analyses of Oligohymenophorea. The main findings are: (1) Utilizing multiple genes is instrumental in improving phylogenetic relationships within class Oligohymenophorea; (2) class Oligohymenophorea is divided into two distinct groups: (i) encompassing the subclass Hymenostomatia, Scuticociliatia, Apostomatia, Astomatia, and Urocentria; and (ii) comprising the subclasses Peniculia and Peritrichia; (3) Revising the phylogenetic placement of the subclass Urocentrida reveals its transitional role as a taxon between Group I and Group II. It emerges as a sister clade to Hymenostomatia; (4) the phylogenetic positions of Astomatia and Apostomatia within the clade ‘SAA’ become clearer; and (5) the order Loxocephalida represents an early member of Scuticociliatia and serves as a potential prototype for this taxonomic group. This study provides more information for understanding the evolutionary relationships within Oligohymenophorea.

## Graphical abstract

We report 97 new sequences, including SSU‐rRNA, ITS1‐5.8S‐ITS2 rRNA, LSU‐rRNA, mitochondrial cytochrome oxidase c subunit I, and mitochondrial small subunit ribosomal RNA genes of 30 oligohymenophorean populations. A comprehensive analysis of class Oligohymenophorea is conducted, encompassing 250 populations. For the first time, phylogenetic analyses of oligohymenophoreans were conducted based on a five‐gene concatenated data set. A comprehensive examination of the subclasses of Oligohymenophorea, based on multiple genes. The concept of Cluster “SAA” is put forth for consideration: Scuticociliatia (including Apostomatia and Astomatia, abbreviated as SAA). A revised phylogenetic classification of the subclass Urocentrida and order Loxocephalida is presented herewith. The evolutionary relationships of class Oligohymenophorea are investigated. This study lays the groundwork for a deep understanding of evolutionary relationships within Oligohymenophorea.

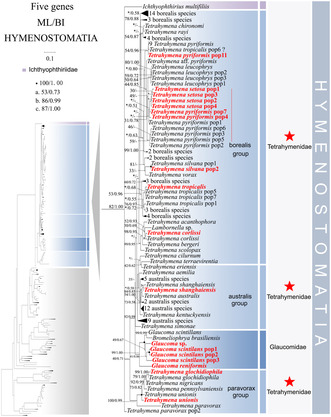

## 1 Introduction

Ciliates are highly differentiated microbial eukaryotes with unique morphological features (e.g., presence of cilia during their life cycle, nuclear dimorphism) that distinguish them from other protozoa (Adl et al. 2012; Chi et al. 2024; Lyu et al. 2024; Lynn 2008; Wu et al. 2023; Zhang, Lu, et al. 2022). Owing to their distinctive characteristics and short lifespan, ciliates are the subjects of investigation in numerous biological disciplines such as evolution, cytology, epigenetics, genetics, and so on (Adl et al. 2012; Arregui et al. 2002; Asghar et al. 2021; Li et al. 2023, 2024; Liu et al. 2022; Wu et al. 2021; Zhang, Tang, et al. 2022). Among ciliates, the class Oligohymenophorea Puytorac et al. (1984), is globally distributed and considered a focal group for research due to its ecological and biological significance (Fan, Hu, et al. 2011; Foissner et al. 2014; Pan et al. 2015). It includes the unicellular model eukaryotes (*Tetrahymena* and *Paramecium*), parasites in fishery (e.g., *Ichthyophthirius* and *Uronema*), important microeukaryotic predators in sewage treatments (e.g., *Vorticella*, *Epistylis*, and *Zoothamnium*), and so on (Kim et al. 2022; Lynn 2008). However, ambiguous phylogenetic relationships within class Oligohymenophorea have hindered in‐depth research in related biological disciplines.

Up to date, the class Oligohymenophorea is composed of seven subclasses: Hymenostomatia, Peniculia, Scuticociliatia, Apostomatia, Peritrichia, Astomatia, and Urocentria (Wang et al. 2021). Due to a lack of sequences, phylogenetic assignments of subclasses Apostomatia, Astomatia, and Urocentria have been less studied. Limited references reported that Apostomatia and Astomatia were consistently within Scuticociliatia (Gao, Katz, and Song 2013; Gao et al. 2016; Poláková, Bourland, and Čepička 2023), and Urocentria has recently been reclassified as a subclass positioned at the base of the sister clade formed by Hymenostomatia and Scuticociliatia (Wang et al. 2021). The placements of Peniculia and Peritrichia were widely studied but presented uncertainty, with three different potential relationships: (1) forming sister clades to each other; (2) Peniculia locates at the base of the Oligohymenophorea; (3) Peritrichia locates at the base of the Oligohymenophorea (Feng et al. 2015; Jiang et al. 2019, 2024; Gentekaki et al. 2017; Wang et al. 2021; Zhang et al. 2023). The placements of the Hymenostomatia and Scuticociliatia also varied in different investigations. For instance, they formed sister clades for each other in phylogenomic or concatenated trees (Feng et al. 2015; Gao, Katz, and Song 2013; Gentekaki et al. 2017; Jiang et al. 2024, 2019; Wang et al. 2021; Zhang et al. 2019) and some SSU‐rRNA gene trees (Campello‐Nunes et al. 2024; Bourland and Strüder‐Kypke 2010; Rataj and Vďačný 2019; Zhang et al. 2019). By contrast, Hymenostomatia was most closely related to Peritrichia in other SSU‐rRNA gene trees (Feng et al. 2015; Li et al. 2006; Poláková, Bourland, and Čepička 2023; Zhang and Vd'ačný 2022).

Not only have placements of subclasses not been resolved, but also relationships within subclasses are still ambiguous. Relationships within the subclass Scuticociliatia remain unresolved because molecular and morphological information cannot be precisely matched (Gao and Katz 2014; Gao et al. 2010; Gao, Katz, and Song 2012; Hao et al. 2022; Huang et al. 2021; Poláková, Čepička, and Bourland 2021; Zhang et al. 2021). Philasterida, as a quintessential species among Scuticociliatia, exhibits a robust monophyletic branching pattern. However, the relationships within their internal families, especially Orchitophryidae, remain a topic of ongoing controversy, mainly because of the discordance between molecular data and morphological characters. Several Orchitophryidae genera do not group together, and the clustering patterns contradict the morphological characteristics (Gao, Katz, and Song 2012; Lynn 2008; Pan et al. 2016; Zhang et al. 2019). Also, the relationships within the non‐monophyletic order Loxocephalida remain largely unresolved. Loxocephalid species are dispersed at the base of Scuticociliatia, Apostomatia, Astomatia, or Urocentria (Antipa, Strüder‐Kypke, and Lynn 2020; Poláková, Čepička, and Bourland 2021; Rataj, Zhang, and Vd'ačný 2022; Zhang and Vďačný 2024). Within subclass Hymenostomatia, there were a huge number of cryptic species in the genus *Tetrahymena*, which appeared identical in morphology but did not form monophyletic clades, posed significant challenges in phylogenetic assignments of *Tetrahymena* species. Meanwhile, the emergence of the paravorax group raised doubts regarding the monophyly of the entire genus *Tetrahymena*. Phylogenetic relationships within Peniculia were mainly studied using SSU‐rRNA gene sequences (Fan, Chen, et al. 2011; Fokin et al. 2006; Pan et al. 2013; Xu, Gao, and Fan 2018). Few previous investigations studied relationships within the subclass Peniculia based on multigene sequences or phylogenomic data, but only limited taxa were included (Liao et al. 2021; Sun et al. 2021; Wang et al. 2021; Weimer, Vďačný, and Wolf 2023).

Phylogenetic studies increasingly rely on multigene sequences or phylogenomic data for more robust results compared to single‐gene markers (Gao et al. 2017; Lian et al. 2023; Sun et al. 2021; Wang et al. 2017; Zhang et al. 2020). Due to the scarcity of species with accessible phylogenomic data, we utilize a multigene tree methodology in this study, covering seven recognized subclasses of Oligohymenophorea. The nuclear rRNA gene, often referred to as the “yardstick” of phylogeny, is one of the earliest and most frequently used genes in phylogenetic studies, providing a rich source of data. Besides the SSU‐rRNA gene, the mitochondrial small subunit ribosomal RNA gene (mtSSU‐rRNA gene) is also a good choice because it has a higher copy number and evolutionary rate (Boore and Brown 1998; Moore 1995; Rand 2003; Zhang et al. 2019). The mitochondrial cytochrome oxidase c subunit I (*cox1*) gene is commonly used as a DNA barcodes to distinguish closely related species (Hajibabaei et al. 2006; Smith et al. 2008). It has also been used to identify species of *Tetrahymena*, *Paramecium* (class Oligohymenophorea), and *Colpoda* (class Colpodea), making it a suitable DNA barcode marker for resolving the interspecific and intraspecific relationships (Lynn and Strüder‐Kypke 2006; Song et al. 2022; Zhao et al. 2013).

Class Oligohymenophorea, particularly the subclasses Hymenostomatia and Scuticociliatia, primarily consists of free‐living forms; however, some groups display parasitic lifestyles or facultatively parasitic lifestyles (Corliss 1970; Pan et al. 2022; Rataj and Vďačný 2020). The blooms of parasitic oligohymenophorean ciliates can lead to economic losses due to parasitism affecting economically valuable animals (Hatai et al. 2001; Liu et al. 2023; Imai et al. 2000). In this study, 97 new sequences of 30 populations from 19 oligohymenophorean species are presented. Furthermore, the research delves into a comprehensive phylogenetic analysis of the Oligohymenophorea class, utilizing three nuclear genes (SSU‐rRNA gene, ITS1‐5.8S‐ITS2 rRNA, LSU‐rRNA) and two mitochondrial genes (*cox1* genes, mtSSU‐rRNA gene), in conjunction with examinations of morphological data. In this study, we expanded the multigene dataset of the Paravorax group within Tetrahymena to address its previous deficiency in molecular information and shed light on the clustering of major lineages within oligohymenophorean ciliates.

## 2 Materials and Methods

### 2.1 Taxon Sampling, Observation, and Terminology

A total of 30 populations representing 19 species from the genera *Tetrahymena*, *Glaucoma*, *Paramecium*, *Uronemita*, and *Cyclidium* were collected in this study. *Tetrahymena* and *Uronemita* species ( *Tetrahymena pyriformis* , *T. setosa* , *T*. *corlissi*, *T. tropicalis* , *T. shanghaiensis* , *T*. *glochidiophila*, *T*. *silvana*, *T*. *unionis*, *Uronema* sp.) were obtained from live specimens of various fish from fish markets in Harbin, China. Remaining species (*Glaucoma* sp., *G. scintillans* , *G. reniformis* , *Cyclidium paravorax*, *C. glaucoma* , *Pseudocohnilembus persalinus*, *Paramecium primaurelia*, *Paramecium sexaurelia*, and *Paramecium caudatum* ) were obtained from several freshwater sites in Harbin, China (Table 1). *Tetrahymena* species were isolated from the skin and surface mucus of the fish, and monoclonal were established in Petri dishes with the host fish tissues added as the nutrient source. Other species were maintained in habitat water and dispensed at room temperature using petri dishes (ca. 25°C). Wheat grains were added to the petri dishes as a nutrient source to promote bacterial growth. Silver carbonate staining (Foissner 1992) was used to reveal the infraciliature. Terminology and classification were mainly according to Gao et al. (2016) and Lynn (2008).

**TABLE 1:** The sampling sites in this study.

Species	Sampling site	Sampling methods	Species	Sampling site	Sampling methods
*Tetrahymena pyriformis*	An international fish market, Daoli district, Harbin (45°44′9″ N; 126°35′41″ E)	Collected from dead *Poecilia reticulata*	*Pseudocohnilembus persalinus*	A freshwater pond, Songbei district, Harbin (45°58′3″ N; 126°35′43″ E)	Collected from water using bottle
*Tetrahymena setosa* pop1	A freshwater aquarium, Hulan district, Harbin (45°52′4″ N; 126°33′4″ E)	Collected from water using bottle	*P. persalinus*	An eutrophic pond, Xiangfang district, Harbin (45°42′40″ N; 126°38′14″ E)	Collected from water using bottle
*Tetrahymena setosa* pop2	An international fish market, Daoli district, Harbin (45°44′9″ N; 126°35′41″ E)	Collected from water using bottle	*Paramecium primaurelia*	An eutrophic pond, Nangang district, Harbin (45°46′5″ N; 126°40′41″ E)	Collected from water using bottle
*Tetrahymena setosa* pop3	An eutrophic pond, Xiangfang district, Harbin (45°42′40″ N; 126°38′14″ E)	Collected from dead *Carassius auratus*	*Paramecium sexaurelia*	An eutrophic pond, Xiangfang district, Harbin (45°43′51″ N; 126°37′55″ E)	Collected from water using bottle
*Tetrahymena setosa* pop4	An international fish market, Daoli district, Harbin (45°44′9″ N; 126°35′41″ E)	Collected from dead *P. reticulata*	*Paramecium caudatum*	An eutrophic pond, Xiangfang district, Harbin (45°42′40″ N; 126°38′14″ E)	Collected from water using bottle
*Tetrahymena silvana*	An international fish market, Daoli district, Harbin (45°44′9″ N; 126°35′41″ E)	Collected from dead *P. reticulata*	*P. caudatum*	A freshwater pond, Songbei district, Harbin (45°58′3″ N; 126°35′43″ E)	Collected from water using bottle
*Tetrahymena tropicalis*	A freshwater aquarium, Hulan district, Harbin (45°52′3″ N; 126°33′2″ E)	Collected from dead *C. auratus*	*Cyclidium paravorax*	A freshwater aquarium, Hulan district, Harbin (45°52′3″ N; 126°33′2″ E)	Collected from water using bottle
*Tetrahymena unionis*	A freshwater aquarium, Hulan district, Harbin (45°52′3″ N; 126°33′2″ E)	Collected from water using bottle	*C. paravorax*	An international fish market, Daoli district, Harbin (45°44′9″ N; 126°35′41″ E)	Collected from dead *Misgurnus anguillicaudatus*
*Tetrahymena corlissi*	An international fish market, Daoli district, Harbin (45°44′9″ N; 126°35′41″ E)	Collected from dead *P. reticulata*	*Cyclidium glaucoma*	An eutrophic pond, Xiangfang district, Harbin (45°42′40″ N; 126°38′14″ E)	Collected from water using bottle
*Tetrahymena shanghaiensis*	A freshwater aquarium, Hulan district, Harbin (45°51′11″ N; 126°10′57″ E)	Collected from water using bottle	*Glaucoma* sp.	A freshwater pond, Songbei district, Harbin (45°58′3″ N; 126°35′43″ E)	Collected from water using bottle
*Tetrahymena glochidiophila*	An international fish market, Daoli district, Harbin (45°44′9″ N; 126°35′41″ E)	Collected from dead *P. reticulata*	*Glaucoma scintillans* pop1	An eutrophic pond, Xiangfang district, Harbin (45°43′51″ N; 126°37′55″ E)	Collected from water using bottle
*Tetrahymena silvana*	A freshwater aquarium, Hulan district, Harbin (45°51′11″ N; 126°10′57″ E)	Collected from water using bottle	*Glaucoma scintillans* pop2	A freshwater aquarium, Hulan district, Harbin (45°52′3″ N; 126°33′2″ E)	Collected from water using bottle
*Uronema* sp.	A freshwater aquarium, Hulan district, Harbin (45°52′4″ N; 126°33′4″ E)	Collected from dead *M. anguillicaudatus*	*Glaucoma scintillans* pop3	An eutrophic pond, Nangang district, Harbin (45°46′5″ N; 126°40′41″ E)	Collected from water using bottle
*Uronema apomarinum*	A freshwater aquarium, Hulan district, Harbin (45°52′4″ N; 126°33′4″ E)	Collected from water using bottle	*Glaucoma reniformis*	An eutrophic pond, Nangang district, Harbin (45°46′41″ N; 126°36′25″ E)	Collected from water using bottle

### 2.2 DNA Extraction, Amplification, and Sequencing

For each species, 5–10 cells were isolated under a stereomicroscope and washed with distilled water to remove potential contaminants and then incubated in non‐nutrient distilled water for 6–12 h to further remove food contamination. Cells were then transferred to an Eppendorf tube with a maximum volume of 5 μL. Total genomic DNA was extracted using the DNeasy & Tissue Kit (Shanghai, QIAGEN, Germany) following the manufacturer's instructions.

Since universal primers are available for five genes involved in this study, the following primers were used for gene amplification. EukA 5′‐AAC CTG GTT GAT CCT GCC AGT‐3′ and EukB, 5′‐TGA TCC TTC TGC AGG TTC ACC TAC‐3′ (Medlin et al. 1988) were used for SSU‐rRNA; 5.8S‐F, 5′‐GTA GGT GAA CCT GCG GAA GGA TCA TTA‐3′ and 5.8S‐R, 5′‐TAC TGA TAT GCT TAA GTT CAG CGG‐3′ (Goggin 1994) for ITS1‐5.8S‐ITS2 rRNA; 5.8S‐F, 5′‐GTA GGT GAA CCT GCG GAA GGA TCA TTA‐3′ and LOR, 5′‐GCT ATC CTG AGR GAA ACT TCG‐3′ (Pawlowski 2000) for LSU‐rRNA; MT‐F, 5′‐TGT GCC AGC AGC CGC GGT AA‐3′ and MT‐R, 5′‐CCC A(C)T ACC A(G)G TAC CTT GTG T‐3′ (van Hoek et al. 2000) for mtSSU‐rRNA; MOU08‐121, 5′‐TCA GGA GCT GCM TTA GCH ACY ATG‐3′ and MOU08‐122, 5′‐TAR TAT AGG ATC MCC WCC ATA AGC‐3′ (Whang, Kang, and Lee 2013) for mitochondrial *cox1* gene. The polymerase chain reaction conditions are summarized in Table 2. Subsequently, PCR product purification was performed using the TIANgel Midi Purification Kit (Beijing, TIANGEN BIOTECH, China), ligated using the PMD 18‐T vector cloning kit (Takara Biomedicals, China), transformed using DH5ɑ Chemically Competent Cell (Shanghai, weidibio, China), and three positive colonies per gene were selected and sent to Sangon Biotech (Shanghai) for sequencing. Only one sequence was finally selected for subsequent data analysis. A total of 28 new SSU‐rRNA genes, 25 ITS1‐5.8S‐ITS2 rRNA genes, 25 LSU‐rRNA genes, 2 mitochondrial *cox 1* genes, and 17 mtSSU‐rRNA gene sequences from 19 Oligohymenophorea species were determined, totaling 97 new sequences (GenBank accession numbers, lengths, and G&C contents are shown in Tables 3 and 4).

**TABLE 2:** Conditions of PCR reactions used for amplification of five molecular markers analyzed in this study.

Molecular marker	PCR program		
	Initial denaturation	Cycling (denaturation, annealing, extension)	Final extension
SSU‐rRNA gene	94°C/5 min	5 cycles: 94°C/30 s, 56°C/105 s, 72°C/120 s 25 cycles: 94°C/30 s, 60°C/105 s, 72°C/120 s	72°C/10 min
ITS1‐5.8S‐ITS2 rRNA gene	94°C/5 min	35 cycles: 94°C/30 s, 58°C/45 s, 72°C/60 s	72°C/10 min
LSU‐rRNA gene	94°C/3 min	35 cycles: 95°C/15 s, 55°C/60 s, 72°C/120 s	72°C/10 min
mtSSU‐rRNA gene	94°C/5 min	5 cycles: 94°C/30 s, 58°C/105 s, 72°C/120 s 25 cycles: 94°C/45 s, 60°C/105 s, 72°C/120 s	72°C/10 min
cox 1 gene	94°C/2 min	30 cycles: 94°C/30 s, 50°C/30 s, 72°C/120 s	72°C/10 min

**TABLE 3:** New sequences of subclass Hymenostomatia were obtained in this study.

Species	Accession numbers	Lengths (bp)	G&C%	Gene markers	Species	Accession numbers	Lengths (bp)	G&C%	Gene markers
*Glaucoma reniformis*	PP254268	1688	43.96	SSU‐rRNA	*Glaucoma scintillans* pop3	PP230703	1687	43.51	SSU‐rRNA
	PP254282	478	42.68	ITS1‐5.8S‐ITS2		PP230704	489	41.92	ITS1‐5.8S‐ITS2 rRNA
	PP254261	961	47.35	LSU‐rRNA		PP254263	964	46.89	LSU‐rRNA
	PP254271	1009	31.52	mtSSU‐rRNA		PP254281	988	32.09	mtSSU‐rRNA
*Tetrahymena silvana* pop2	MW194101	1821	28.50	COX 1	*Tetrahymena pyriformis* pop7	OR878270	1670	43.17	SSU‐rRNA
*Tetrahymena pyriformis* pop13	MW194102	689	27.58	COX 1		PP229189	457	40.04	ITS1‐5.8S‐ITS2 rRNA
*Tetrahymena pyriformis* pop4	OR878269	1683	43.37	SSU‐rRNA		PP229191	944	47.35	LSU‐rRNA
	PP213444	455	39.34	ITS1‐5.8S‐ITS2 rRNA	*Tetrahymena tropicalis*	OR865347	1681	43.01	SSU‐rRNA
	PP213445	948	46.94	LSU rDNA		PP211944	456	41.23	ITS1‐5.8S‐ITS2 rRNA
*Tetrahymena setosa* pop2	PP212013	1691	43.11	SSU rDNA		PP211947	971	46.55	LSU‐rRNA
	PP213448	460	40.22	ITS1‐5.8S‐ITS2 rRNA	*Tetrahymena shanghaiensis*	PP230698	1702	43.24	SSU‐rRNA
	PP213446	958	46.97	LSU‐rRNA		PP230699	465	40.43	ITS1‐5.8S‐ITS2 rRNA
	PP213447	1028	32.20	mtSSU‐rRNA		PP254262	962	46.67	LSU‐rRNA
*Tetrahymena setosa* pop3	OR461621	1697	43.37	SSU‐rRNA		PP254284	983	28.08	mtSSU‐rRNA
	PP229186	462	40.26	ITS1‐5.8S‐ITS2 rRNA	*Tetrahymena glochidiophila*	OR616813	1684	43.17	SSU‐rRNA
	PP229188	952	47.06	LSU‐rRNA		PP254283	467	40.47	ITS1‐5.8S‐ITS2 rRNA
	PP229187	1027	32.52	mtSSU‐rRNA		PP254264	943	45.71	LSU‐rRNA
*Tetrahymena unionis*	PP439292	1659	43.40	SSU‐rRNA		PP230930	1016	32.09	mtSSU‐rRNA
	PP439293	466	40.56	ITS1‐5.8S‐ITS2 rRNA	*Tetrahymena corlissi*	OR858827	1675	43.34	SSU‐rRNA
	PP440017	960	46.35	LSU‐rRNA		PP217228	458	40.83	ITS1‐5.8S‐ITS2 rRNA
	PP439303	998	31.56	mtSSU‐rRNA		PP229190	954	45.70	LSU‐rRNA
*Tetrahymena setosa* pop1	OR878268	1679	43.18	SSU‐rRNA	*Glaucoma* sp.	OR878267	1678	43.62	SSU‐rRNA
*Glaucoma scintillans* pop1	PP230915	1667	43.25	SSU‐rRNA		PP230916	496	42.74	ITS1‐5.8S‐ITS2 rRNA
	PP230917	496	43.35	ITS1‐5.8S‐ITS2 rRNA		QR878272	963	46.11	LSU‐rRNA
	OR878271	961	46.72	LSU‐rRNA	*Tetrahymena setosa* pop4	OR616814	1688	43.01	SSU‐rRNA
*Glaucoma scintillans* pop2	PP230912	1675	43.34	SSU‐rRNA		PP213055	460	39.78	ITS1‐5.8S‐ITS2 rRNA
	PP230914	501	42.91	ITS1‐5.8S‐ITS2 rRNA		PP213442	952	47.27	LSU‐rRNA
	PP230913	962	46.67	LSU‐rRNA		PP213443	1021	32.03	mtSSU‐rRNA
	PP254280	970	30.31	mtSSU‐rRNA					

**TABLE 4:** New sequences of subclass Scuticociliatia and Peniculia were obtained in this study.

Species	Accession numbers	Lengths (bp)	G&C%	Gene markers	Species	Accession numbers	Lengths (bp)	G&C%	Gene markers
*Uronema* sp. pop1	PP254269	1689	43.22	SSU‐rRNA	*Cyclidium paravorax*	OR466404	1700	45.82	SSU‐rRNA
	PP254287	448	31.25	ITS1‐5.8S‐ITS2 rRNA		PP254277	526	34.79	ITS1‐5.8S‐ITS2 rRNA
	PP254267	958	43.84	LSU‐rRNA		PP230948	989	50.46	LSU‐rRNA
	PP254272	942	25.27	mtSSU‐rRNA		PP254273	930	24.62	mtSSU‐rRNA
*Uronema* sp. pop2	PP254270	1687	43.27	SSU‐rRNA	*Paramecium primaurelia*	PP237001	1693	44.60	SSU‐rRNA
	PP254286	450	32.22	ITS1‐5.8S‐ITS2 rRNA		PP237006	434	37.56	ITS1‐5.8S‐ITS2 rRNA
	PP254266	962	43.87	LSU‐rRNA		PP237007	981	46.79	LSU‐rRNA
	PP254276	937	24.55	mtSSU‐rRNA		PP238398	983	34.99	mtSSU‐rRNA
*Uronema* sp. pop3	PP254274	1673	43.21	SSU rDNA	*Paramecium sexaurelia*	PP237013	1681	44.31	SSU‐rRNA
	PP254285	448	31.92	ITS1‐5.8S‐ITS2 rRNA		PP237220	428	37.38	ITS1‐5.8S‐ITS2 rRNA
	PP254265	952	44.22	LSU‐rRNA		PP237219	981	46.79	LSU‐rRNA
	PP254275	940	24.89	mtSSU‐rRNA		PP238400	973	35.05	mtSSU‐rRNA
*Cyclidium glaucoma*	PP230946	1681	43.43	SSU‐rRNA	*Paramecium caudatum* pop1	PP236973	1677	44.60	SSU‐rRNA
	PP254278	502	36.65	ITS1‐5.8S‐ITS2 rRNA		PP236974	427	35.83	ITS1‐5.8S‐ITS2 rRNA
	PP230947	984	50.41	LSU‐rRNA		PP236975	982	45.93	LSU‐rRNA
*Pseudocohnilembus persalinus* pop3	OR858824	1719	45.49	SSU‐rRNA		PP238396	974	32.65	mtSSU‐rRNA
*Pseudocohnilembus persalinus* pop4	OR878266	1712	45.50	SSU‐rRNA	*Paramecium caudatum* pop3	PP236958	1661	44.07	SSU‐rRNA
*Cyclidium paravorax*	OR857505	1680	45.48	SSU‐rRNA		PP236966	429	35.66	ITS1‐5.8S‐ITS2 rRNA
	PP254279	506	33.99	ITS1‐5.8S‐ITS2 rRNA		PP236968	976	46.11	LSU‐rRNA
	OR865348	993	50.65	LSU‐rRNA		PP238395	940	33.09	mtSSU‐rRNA

### 2.3 Datasets and Alignments

**FIGURE 1:**
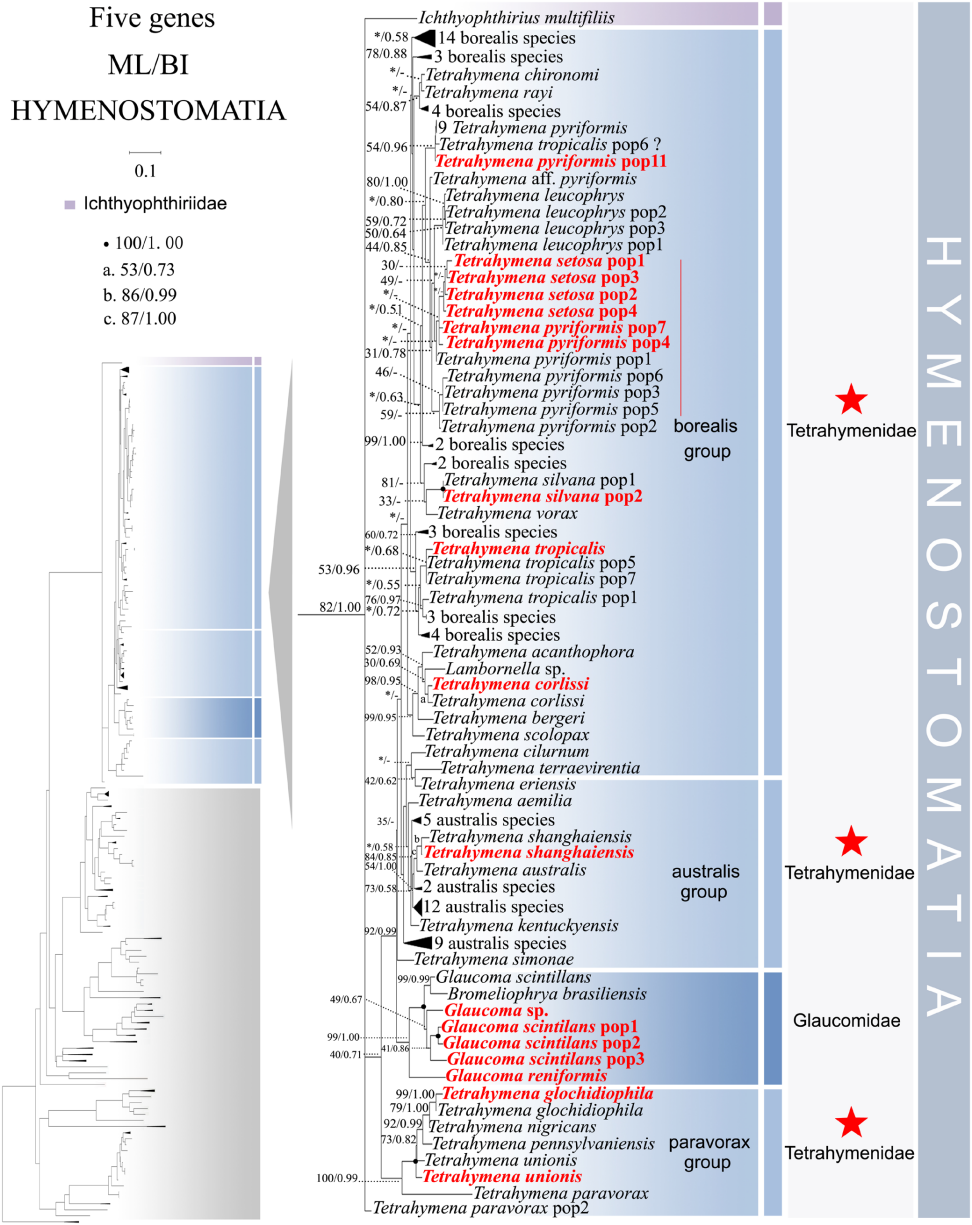
Phylogenetic tree relationships within the subclass Hymenostomatia based on the concatenated genes (SSU‐rRNA, ITS1‐5.8S‐ITS2 rRNA, LSU‐rRNA, mtSSU‐rRNA, and *cox 1* genes). Owing to its large size of the image, the phylogenetic tree has been segmented into two parts, labeled as ‘Hymenostomatia’ and ‘Scuticociliatia, Astomatia, Apostomatia, Urocentria, Peniculia, Peritrichia’. A comprehensive view of relationships within the class Oligohymenophorea is presented in the lower left of the image, while the colored section represents a portion of the original image (subclass Hymenostomatia). Newly sequenced species in this study are in red. The supports for nodes are indicated as follows: ML bootstraps/BI posterior probability. The symbol ‘‐’ denotes inconsistency in topology between the Bayesian and ML trees. Fully supported (100%/1.00) clades are marked with solid circles. ‘*’ at nodes indicates the support values < 30%/0.5 (ML/BI). The red five‐pointed star, red line specifically denotes the group that is the central focus of this study. The scale bar corresponds to 0.1 expected substitutions per site. The long clades has been shortened, as shown by ‘//’, and the other clades are drawn to scale.

**FIGURE 2:**
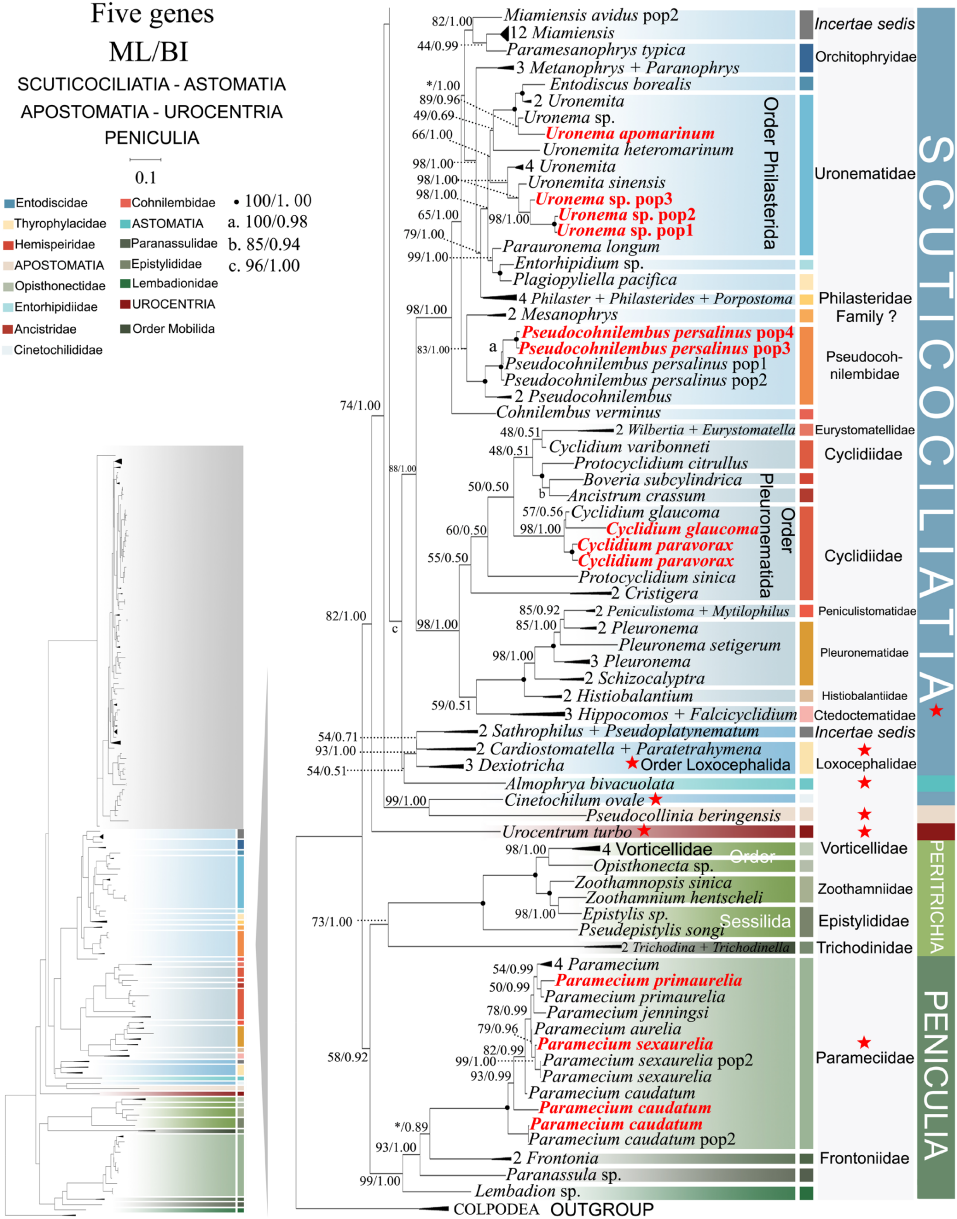
Phylogenetic relationships among subclass Scuticociliatia, Astomatia, Apostomatia, Urocentria, Peniculia, Peritrichia based on the concatenated genes (SSU‐rRNA, ITS1‐5.8S‐ITS2 rRNA, LSU‐rRNA, mtSSU‐rRNA and *cox 1* genes). Owing to its large size of the image, the phylogenetic tree has been segmented into two parts, labeled as ‘Hymenostomatia’ and ‘Scuticociliatia, Astomatia, Apostomatia, Urocentria, Peniculia, Peritrichia’. A comprehensive view of relationships within the class Oligohymenophorea is presented in the lower left of the image, while the colored section represents a portion of the original image (subclass Scuticociliatia, Astomatia, Apostomatia, Urocentria, Peniculia, Peritrichia). Newly sequenced species in this study are in red. The supports for nodes are indicated as follows: ML bootstraps/BI posterior probability. The symbol ‘‐’ denotes inconsistency in topology between the Bayesian and ML trees. Fully supported (100%/1.00) clades are marked with solid circles. ‘*’ at nodes indicates the support values < 30%/0.5 (ML/BI). The red five‐pointed star specifically denotes the group that is the central focus of this study. The scale bar corresponds to 0.1 expected substitutions per site. The long clades has been shortened, as shown by ‘//’, and the other clades are drawn to scale.

**FIGURE 3:**
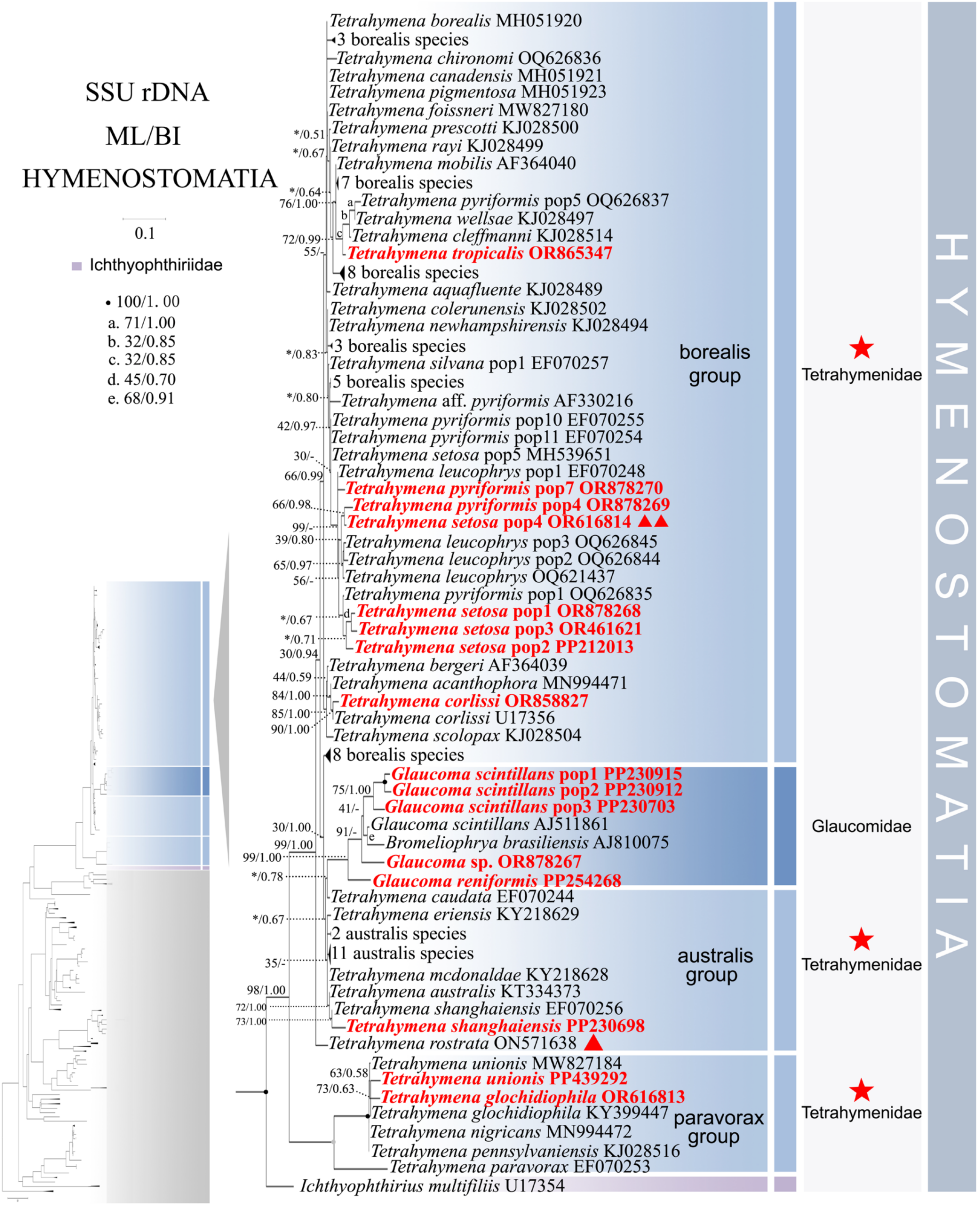
Phylogenetic relationships within the subclass Hymenostomatia based on the SSU‐rRNA gene. Owing to its large size of the image, the phylogenetic tree has been segmented into two parts, labeled as ‘Hymenostomatia’ and ‘Scuticociliatia, Astomatia, Apostomatia, Urocentria, Peniculia, Peritrichia’. A comprehensive view of relationships within the class Oligohymenophorea is presented in the lower left of the image, while the colored section represents a portion of the original image (subclass Hymenostomatia). Newly sequenced species in this study are in red. The support for nodes are indicated as follows: ML bootstraps/BI posterior probability. ‘‐’ indicate mismatch in topology between Bayesian and ML trees. Fully supported (100%/1.00) clades are marked with solid circles. ‘*’ at nodes indicates the support values < 30%/0.5 (ML/BI). The long clades has been shortened, as shown by ‘//’, and the other clades are drawn to scale. The red five‐pointed star, triangle specifically denotes the group that is the central focus of this study. The scale bar corresponds to 0.1 expected substitutions per site.

**FIGURE 4:**
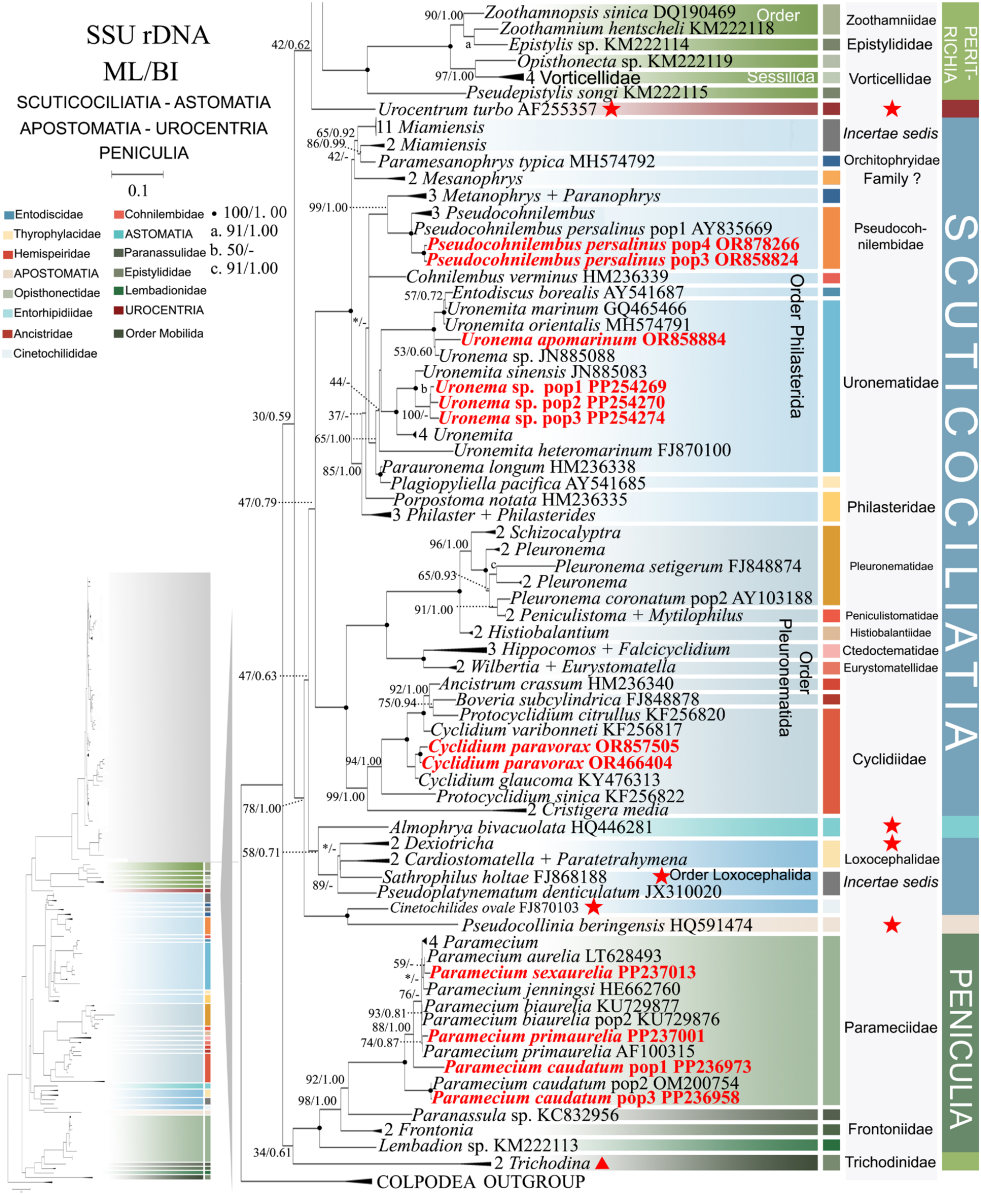
Phylogenetic relationships among subclass Scuticociliatia, Astomatia, Apostomatia, Urocentria, Peniculia, Peritrichia based on the SSU‐rRNA gene. Owing to its large size of the image, the phylogenetic tree has been segmented into two parts, labeled as ‘Hymenostomatia’ and ‘Scuticociliatia, Astomatia, Apostomatia, Urocentria, Peniculia, Peritrichia’. A comprehensive view of relationships within the class Oligohymenophorea is presented in the lower left of the image, while the colored section represents a portion of the original image (subclass Scuticociliatia, Astomatia, Apostomatia, Urocentria, Peniculia, Peritrichia). Newly sequenced species in this study are in red. The supports for nodes are indicated as follows: ML bootstraps/BI posterior probability. ‘‐’ indicate mismatch in topology between Bayesian and ML trees. Fully supported (100%/1.00) clades are marked with solid circles. ‘*’ at nodes indicates the support values < 30%/0.5 (ML/BI). The long clades has been shortened, as shown by ‘//’, and the other clades are drawn to scale. The red five‐pointed star and triangle specifically denote the group that is the main focus of this study. The scale bar corresponds to 0.1 expected substitutions per site.

**FIGURE 5:**
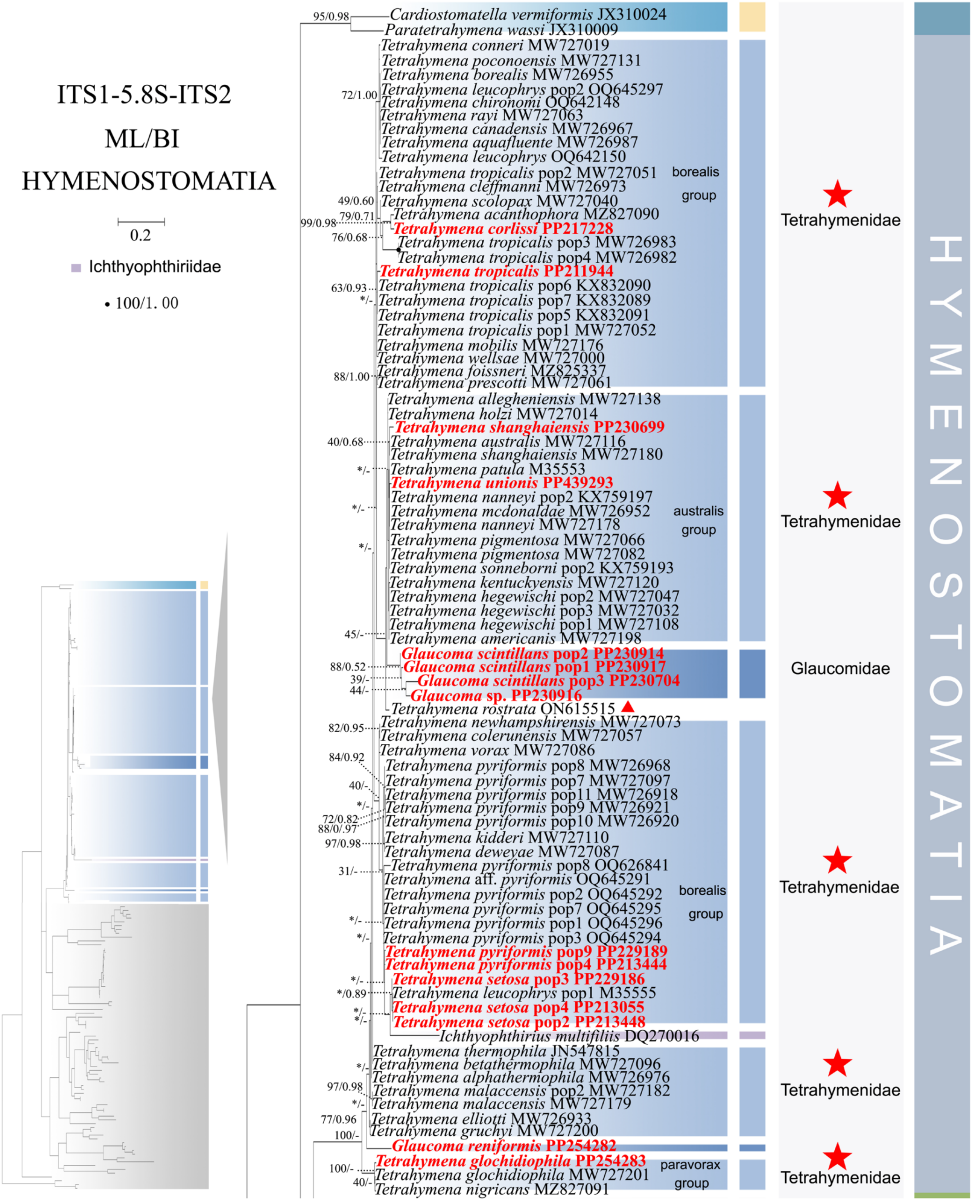
Phylogenetic relationships within the subclass Hymenostomatia based on the ITS1‐5.8S‐ITS2 rRNA gene. Owing to its large size of the image, the phylogenetic tree has been segmented into two parts, labeled as ‘Hymenostomatia’ and ‘Scuticociliatia, Astomatia, Apostomatia, Urocentria, Peniculia, Peritrichia’. A comprehensive view of relationships within the class Oligohymenophorea is presented in the lower left of the image, while the colored section represents a portion of the original image (subclass Hymenostomatia). Newly sequenced species in this study are in red. The supports for nodes are indicated as follows: ML bootstraps/BI posterior probability. ‘‐’ indicate mismatch in topology between Bayesian and ML trees. Fully supported (100%/1.00) clades are marked with solid circles. ‘*’ at nodes indicates the support values < 30%/0.5 (ML/BI). The long clades has been shortened, as shown by ‘//’, and the other clades are drawn to scale. The red five‐pointed star, triangle specifically denotes the group that is the central focus of this study. The scale bar corresponds to 0.2 expected substitutions per site.

**FIGURE 6:**
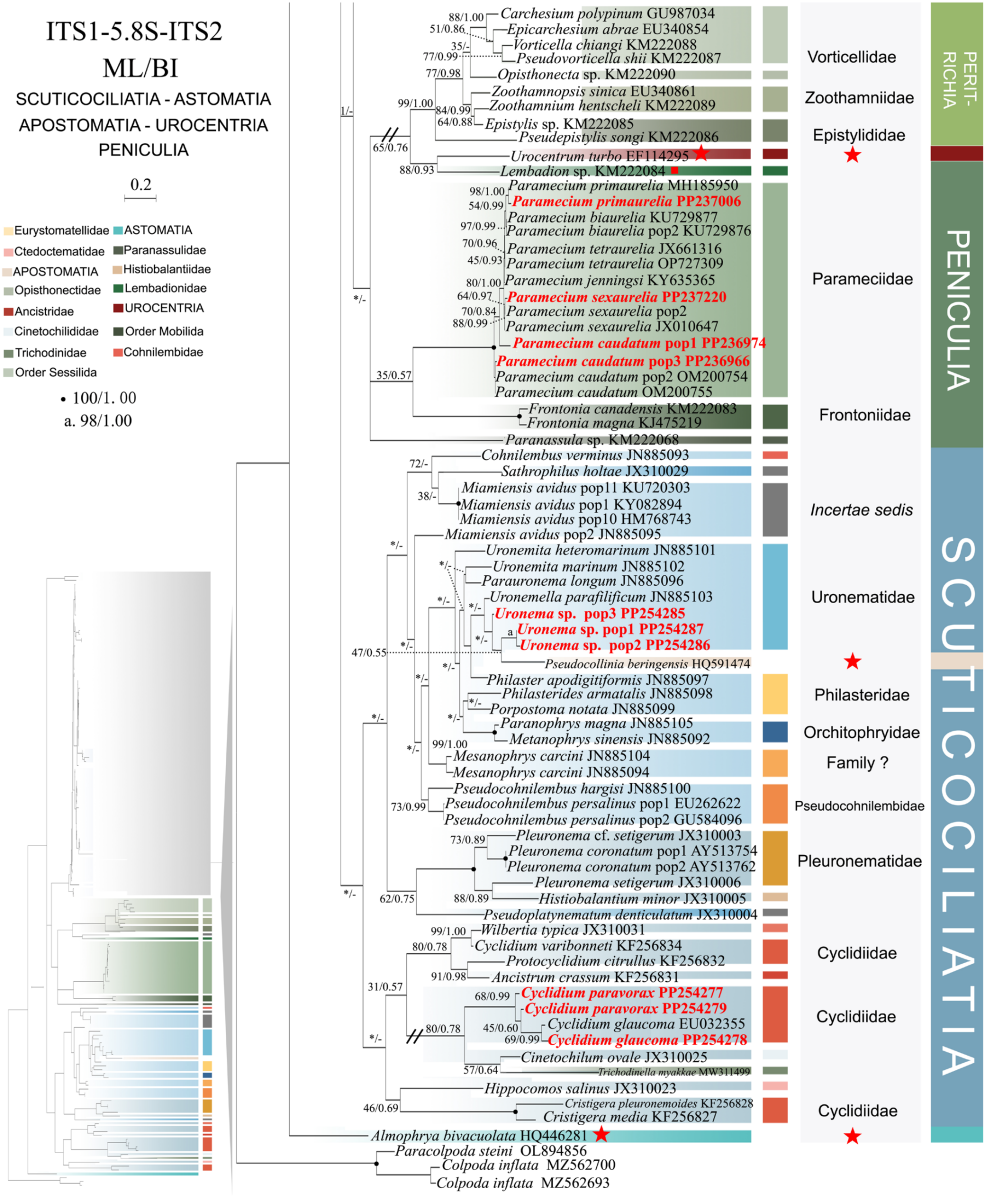
Phylogenetic relaitionships among subclass Scuticociliatia, Astomatia, Apostomatia, Urocentria, Peniculia, Peritrichia based on the ITS1‐5.8S‐ITS2 rRNA gene. Owing to its large size of the image, the phylogenetic tree has been segmented into two parts, labeled as ‘Hymenostomatia’ and ‘Scuticociliatia, Astomatia, Apostomatia, Urocentria, Peniculia, Peritrichia’. A comprehensive view of relationships within the class Oligohymenophorea is presented in the lower left of the image, while the colored section represents a portion of the original image (subclass Scuticociliatia, Astomatia, Apostomatia, Urocentria, Peniculia, Peritrichia). Newly sequenced species in this study are in red. The supports for nodes are indicated as follows: ML bootstraps/BI posterior probability. ‘‐’ indicate mismatch in topology between Bayesian and ML trees. Fully supported (100%/1.00) clades are marked with solid circles. ‘*’ at nodes indicates the support values < 30%/0.5 (ML/BI). The long clades has been shortened, as shown by ‘//’, and the other clades are drawn to scale. The red five‐pointed star, square specifically denotes the group that is the central focus of this study. The scale bar corresponds to 0.2 expected substitutions per site.

**FIGURE 7:**
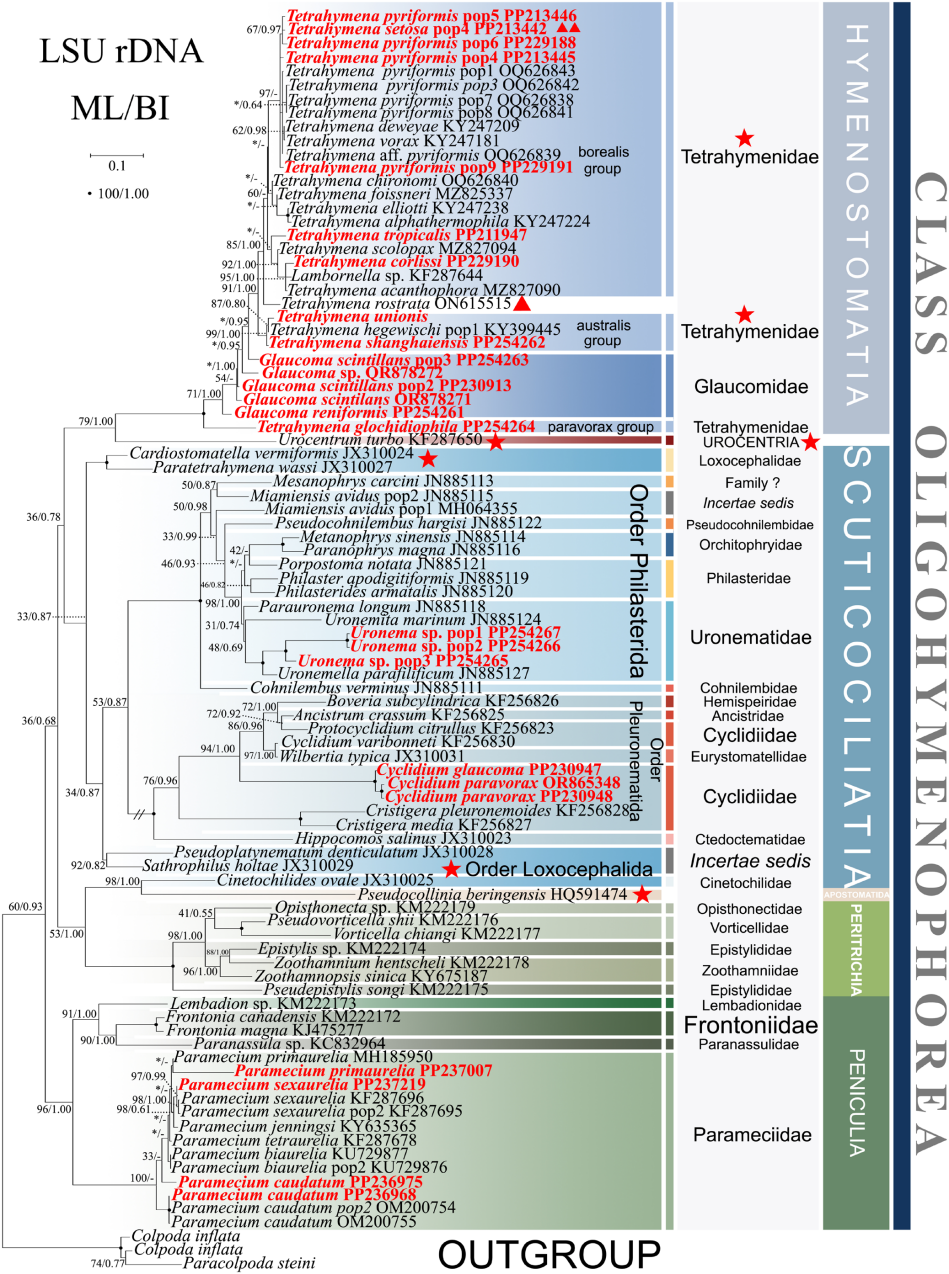
Phylogenetic tree of class Oligohymenophorea based on the LSU‐rRNA gene. Newly sequenced species in this study are in red. The supports for nodes are indicated as follows: ML bootstraps/BI posterior probability. ‘‐’ indicate mismatch in topology between Bayesian and ML trees. Fully supported (100%/1.00) clades are marked with solid circles. ‘*’ at nodes indicates the support values < 50%/0.5 (ML/BI). The red five‐pointed star, double triangle specifically denotes the group that is the central focus of this study. The scale bar corresponds to 0.1 expected substitutions per site.

**FIGURE 8:**
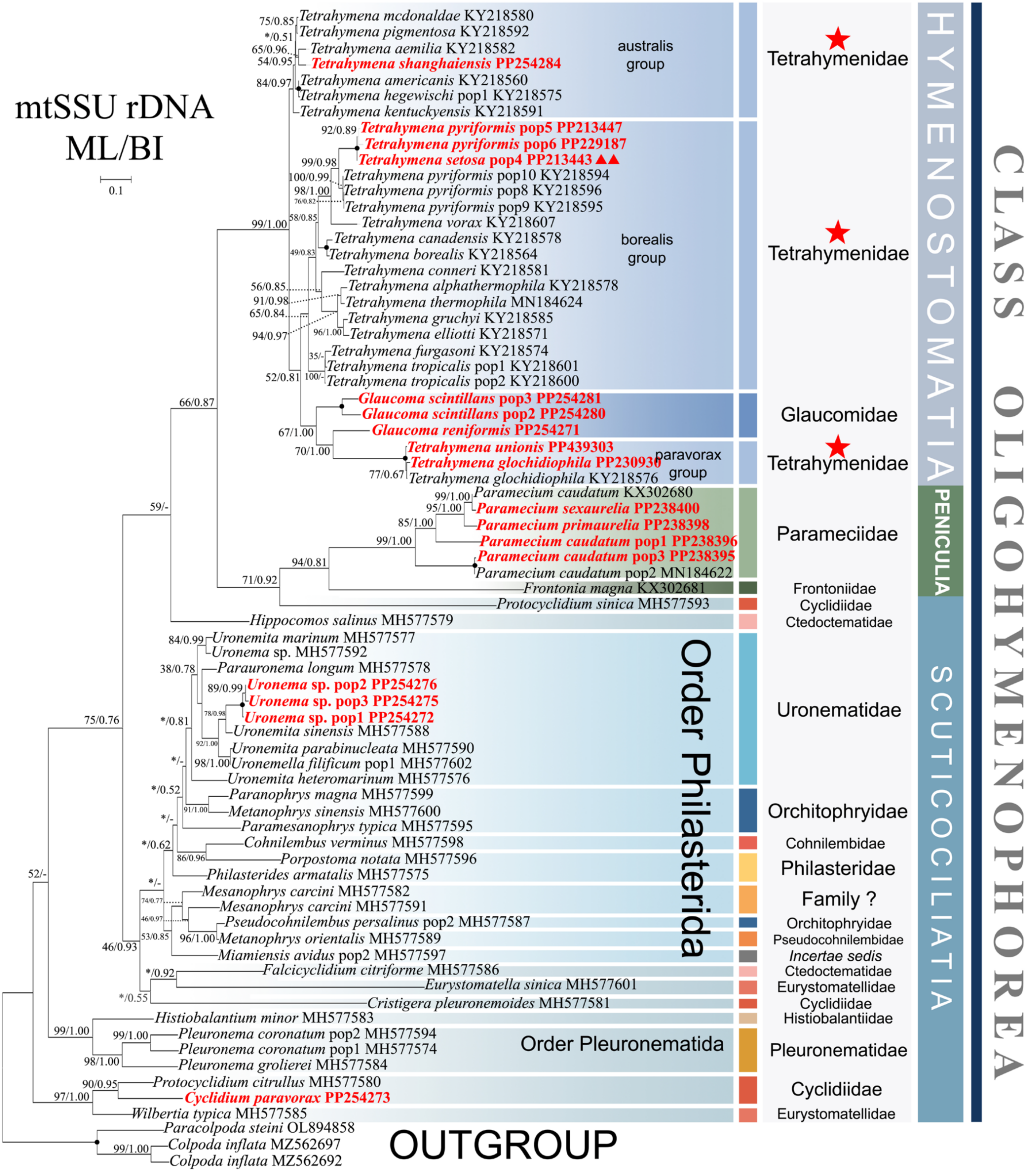
Phylogenetic tree of class Oligohymenophorea based on the mtSSU‐rRNA gene. Newly sequenced species in this study are in red. The supports for nodes are indicated as follows: ML bootstraps/BI posterior probability. ‘‐’ indicate mismatch in topology between Bayesian and ML trees. Fully supported (100%/1.00) clades are marked with solid circles. ‘*’ at nodes indicates the support values < 50%/0.5 (ML/BI). The red five‐pointed star, double triangle specifically denotes the group that is the central focus of this study. The scale bar corresponds to 0.2 expected substitutions per site.

**FIGURE 9:**
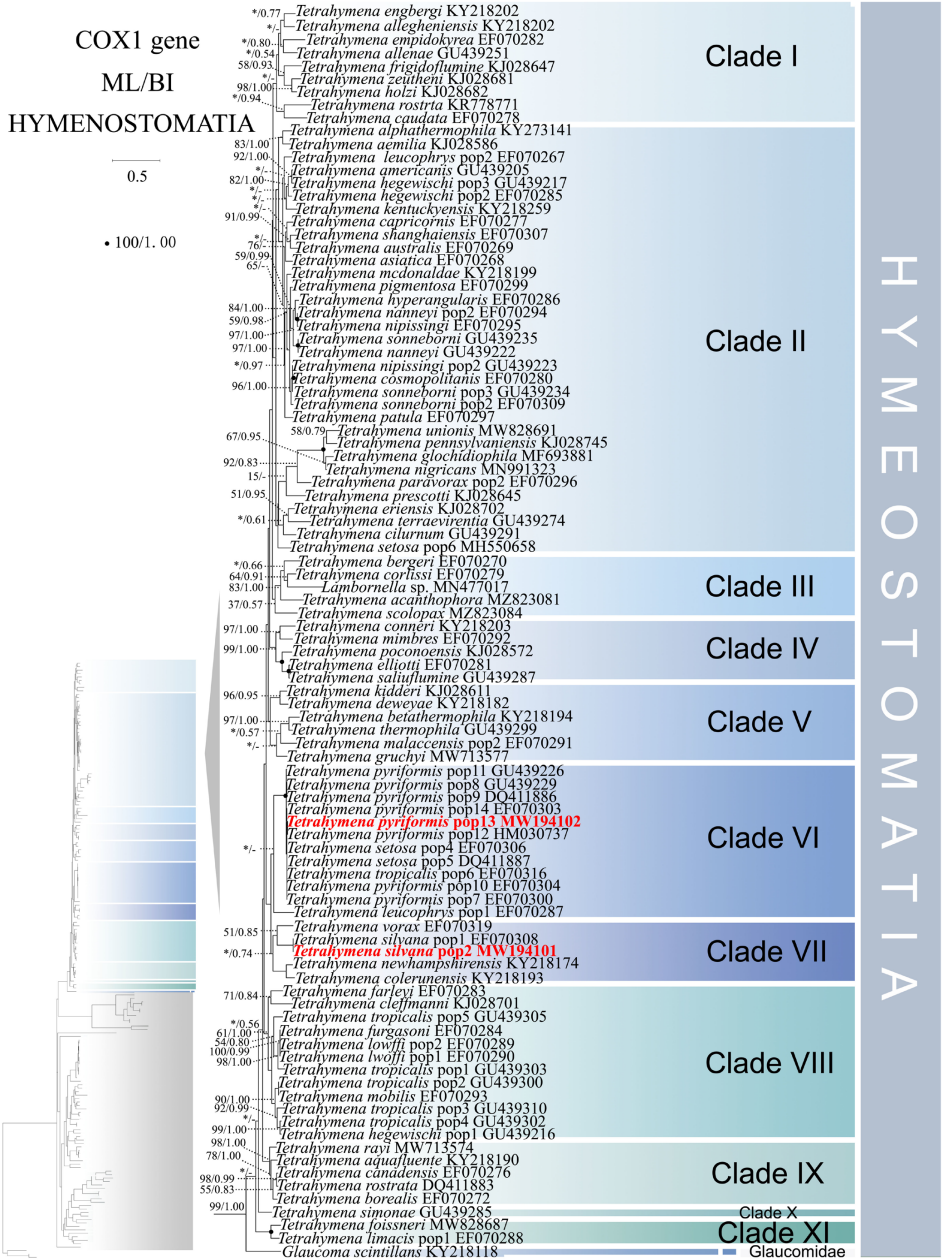
Phylogenetic tree of subclass Hymenostomatia based on the *cox 1* gene. Owing to its large size of the image, the phylogenetic tree has been segmented into two parts, labeled as ‘Hymenostomatia’ and ‘Scuticociliatia, Astomatia, Apostomatia, Urocentria, Peniculia, Peritrichia’. A comprehensive view of relationships within the class Oligohymenophorea is presented in the lower left of the image, while the colored section represents a portion of the original image (subclass Hymenostomatia). Newly sequenced species in this study are in red. The supports for nodes are indicated as follows: ML bootstraps/BI posterior probability. ‘‐’ indicate mismatch in topology between Bayesian and ML trees. Fully supported (100%/1.00) clades are marked with solid circles. ‘*’ at nodes indicates the support values < 50%/0.5 (ML/BI). The red five‐pointed star specifically denotes the group that is the central focus of this study. The scale bar corresponds to 0.5 expected substitutions per site.

**FIGURE 10:**
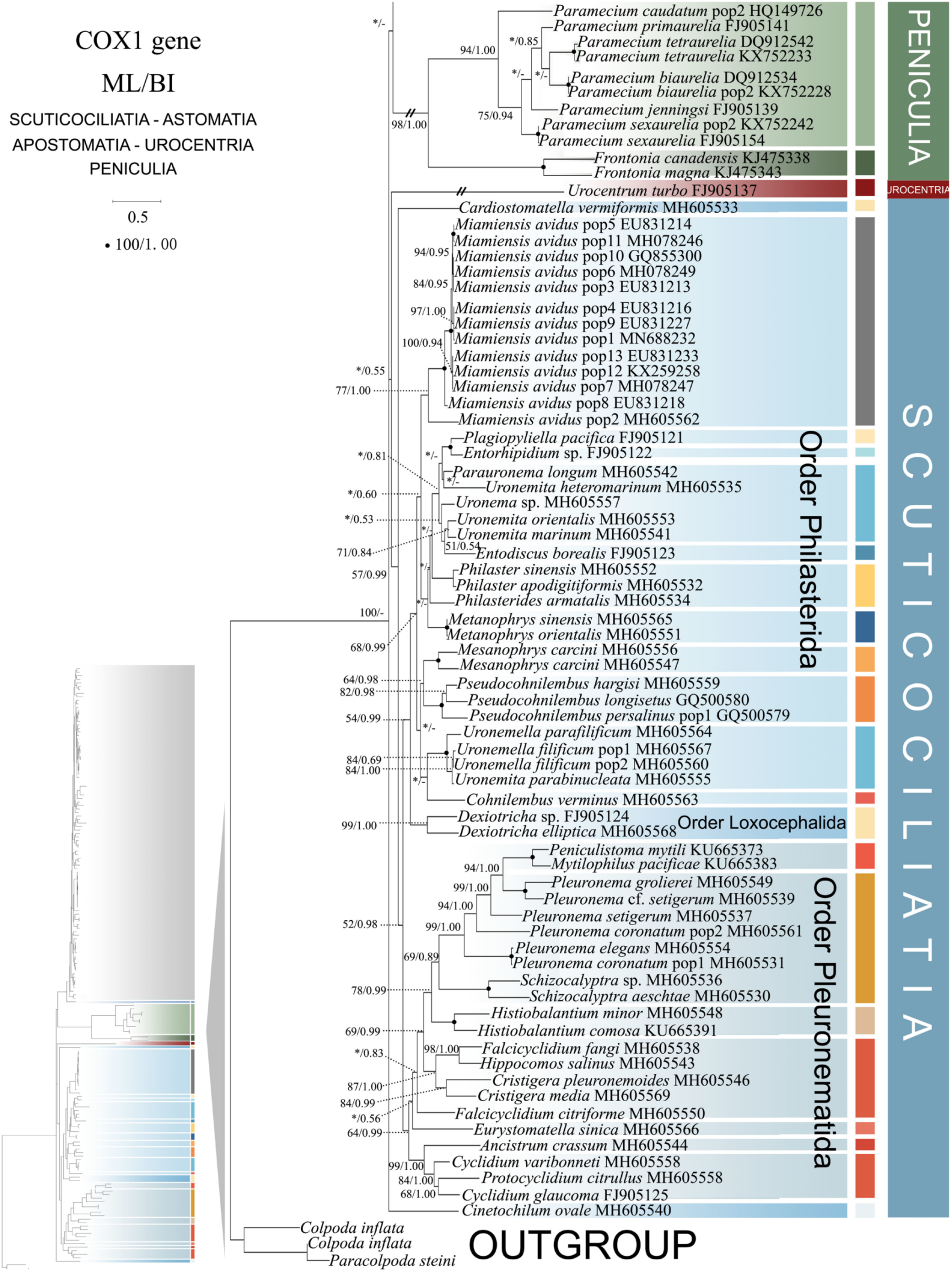
Phylogenetic tree of subclass Scuticociliatia, Astomatia, Apostomatia, Urocentria, Peniculia, Peritrichia based on the *cox 1* gene. Owing to its large size of the image, the phylogenetic tree has been segmented into two parts, labeled as ‘Hymenostomatia’ and ‘Scuticociliatia, Astomatia, Apostomatia, Urocentria, Peniculia, Peritrichia’. A comprehensive view of relationships within the class Oligohymenophorea is presented in the lower left of the image, while the colored section represents a portion of the original image (subclass Scuticociliatia, Astomatia, Apostomatia, Urocentria, Peniculia). Newly sequenced species in this study are in red. The supports for nodes are indicated as follows: ML bootstraps/BI posterior probability. ‘‐’ indicate mismatch in topology between Bayesian and ML trees. Fully supported (100%/1.00) clades are marked with solid circles. ‘*’ at nodes indicates the support values < 50%/0.5 (ML/BI). The scale bar corresponds to 0.5 expected substitutions per site.

The dataset includes representative taxa from all seven subclasses of the class Oligohymenophorea. In addition to newly sequenced data, sequences from GenBank were incorporated (Figures 1, 2, 3, 4, 5, 6, 7, 8, 9, 10). Low‐quality sequences were removed, such as those with questionable morphological information, unknown source, or too short in length. The *cox 1* nucleotide sequences were translated into amino acid sequences using the genetic code for “Protozoan Mitochondrial” and subsequently aligned using MEGA 7 (Kumar, Stecher, and Tamura 2016). Seven datasets were constructed. (i) SSU‐rRNA gene: SSU‐rRNA gene sequences included representatives of 221 taxa (1752 positions); (ii) ITS1‐5.8s‐ITS2: ITS1‐5.8S‐ITS2 rRNA sequences of 159 taxa (542 positions); (iii) LSU‐rRNA gene sequences of 92 taxa (990 positions); (iv) mtSSU‐rRNA gene sequences of 73 taxa (1037 positions); (v) *cox 1* gene sequences of 173 taxa (1821 positions); (vi) *cox 1* amino‐acid code sequences of 173 taxa (607 positions); (vii) Five genes: concatenation of the aligned SSU‐rRNA, ITS1‐5.8S‐ITS2 rRNA, LSU‐rRNA, mtSSU‐rRNA, and *cox 1* gene from datasets i–v (250 taxa, 6142 positions). All the sequences were aligned using MUSCLE. The ambiguous regions in the results were excluded in BioEdit 7.0.1 (Hall 1999). For all datasets, two colpodean species ( *Colpoda inflata* and *Paracolpoda steini*) were selected as outgroups.

### 2.4 Phylogenetic Analyses

Confidence scores calculated by GUIDANCE were used to identify and remove ambiguous columns, followed by manual trimming of both ends of the alignments using Bioedit 7.0.1. (Hall 1999). Maximum likelihood (ML) analyses were constructed by RAxML‐HPC2 v8.2.12 (Stamatakis 2014) and Bayesian inference (BI) analyses by MrBayes v3.2.7a (Ronquist et al. 2012), both on the CIPRES Science Gateway (URL: http://www.phylo.org/sub_sections/portal). The most appropriate models for ML and BI analyses of the SSU‐rRNA gene, ITS1‐5.8S‐ITS2, LSU‐rRNA gene, mtSSU‐rRNA, *cox1* gene, and five genes were determined using Modeltest v3.4 (Posada and Crandall 1998) and the MrModeltest v.2.2 program (Nylander 2004), respectively. The GTR + I + G model was selected for all nucleotide datasets by the Akaike Information Criterion (AIC) in MrModeltest 2.2 (Nylander et al. 2004). The most appropriate model for cox 1 amino acid sequences was mtREV‐F, selected under AIC by ProtTest v3.3 (Adachi et al. 2000; Darriba et al. 2011). A rapid bootstrap with 1000 non‐parametric bootstrap replicates was used for the ML analyses. Four chains were run for 10,000,000 generations, with sampling every 100 generations. The first 25% of sampled trees were discarded as burn‐in. MEGA 7 (Kumar, Stecher, and Tamura 2016) was utilized to visualize tree topologies.

### 2.5 Sequence Analyses and Putative Secondary Structure Modeling

The ITS2 secondary structures of 6 ciliate species were predicted from the *Tetrahymena rostrata* (KY864168) model using the default settings of the Mfold website (http://www.unafold.org/mfold/applications/rna‐folding‐form.php) (Weimer, Vďačný, and Wolf 2023; Zuker 2003). The 5′ and 3′ ends of the ITS2 sequences were determined via Rfam (available on the web http://www.sanger.ac.uk/Software/Rfam/; Griffiths‐Jones et al. 2003), and the exact locations of the 5′ and 3′ ends were further identified through the *Tetrahymena rostrata* (KY864168) model. The predicted structures were visualized using Rnaviz 2.0 (de Rijk and de Wachter 1997).

## 3 Results

### 3.1 Phylogenetic Trees Inferred From Concatenated Dataset (Figures 1, 2, and 12)

As ML and BI trees exhibited similar topologies, only ML trees and corresponding support values from both methods are shown (Figures 1 and 2). Within the class Oligohymenophorea, a monophyletic branching pattern is observed, featuring two main groups: (I) subclasses Hymenostomatia and Scuticociliatia (including Apostomatia and Astomatia, abbreviated as SAA) and Urocentria (82% ML, 1.00 BI); (II) subclasses Peniculia and Peritrichia (58% ML, 0.92 BI) (Figures 1 and 2). The subclass Hymenostomatia comprises three families: Tetrahymenidae, Glaucomidae, and Ichthyophthiriidae (Figure 1). Notably, the family Ichthyophthiriidae, represented by a single species (*Ichthyophthirius multifiliis*), clusters with the Tetrahymenidae + Glaucomidae clade (supported by 82% ML, 1.00 BI). Glaucomidae is positioned between the borealis group and the australis group within the three groups (borealis, australis, and paravorax) of Tetrahymenidae. Apostomatia and Astomatia, each represented by one sequence, cluster at the periphery of Scuticociliatia, leading to the conclusion that Scuticociliatia is polyphyletic (Figure 2). Therefore, we suggest a conceptual framework that consolidates these three entities into a unified cluster termed “SSA” (the Scuticociliatia includes both the Apostomatia and Astomatia).

Within subclass Scuticociliatia, the orders Pleuronematida and Philasterida are observed as monophyletic. Pleuronematida and Philasterida cluster together to form a clade that is sister to order Loxcephalida + Astomatia. Three *Uronema* sp. populations form a sister group with *Uronemita sinensis* (98% ML, 1.00 BI). Four *P. persalinus* populations, including two newly sequenced populations (Pop3–Pop4), cluster together as a sister group (100% ML, 1.00 BI) to *P. hargisi* and *P. longisetus* with full support. Two newly sequenced *C. paravorax* populations also cluster together (100% ML, 1.00 BI), and subsequently form a sister group with *C. glaucoma* (98% ML, 1.00 BI). *Cinetochilides ovale* (Loxcephalida) and *Pseudocollinia beringensis* (Apostomatia) group with strong support (99% ML, 1.00 BI), and subsequently form a sister clade to Hymenostomatia and Scuticociliatia (74% ML, 1.00 BI). *Urocentrum turbo* (Urocentria) falls into the basal position of group I, making it a sister clade to Hymenostomatia and “SAA” (82% ML, 1.00 BI). The subclass Peniculia is represented by four families and four genera (*Paramecium*, *Lambadion*, *Frontonia*, and *Paranassula*) forms a strongly supported monophyletic clade (99% ML, 1.00 BI) and then group with subclass Peritrichia. Subclass Peritrichia comprises two orders and five families. The order Mobilida (Trichodinidae) is clustered adjacent to the order Sessilida (Vorticellidae, Opisthonectidae, Zoothamniidae, and Epistylididae) as a sister clade. Within subclass Hymenostomatia, *Tetrahymena* species are distributed into three groups, with the majority situated in the borealis group. Newly sequenced three *Tetrahymena leucophrys* populations (80% ML, 1.00 BI) and four *T. setosa* populations clusters together, respectively. *T. pyriformis* , including seven newly sequenced and nine previous populations, are divided into two groups. One Harbin population of *Tetrahymena shanghaiensis* group with *T. shanghaiensis* with strong support (86% ML, 0.99 BI). One Harbin population of *T*. *silvana* cluster with *T*. *silvana* with full support. *Tetrahymena unionis* aligns closely with other paravorax species, with strong support. Two *T. glochidiophila* species clusters as a sister group (99% ML, 1.00 BI). The clade of *Glaucoma reniformis* acts as a sister to the clade containing all Glaucomidae species, with substantial support (99% ML, 1.00 BI). Furthermore, *Glaucoma* sp. clusters with *G. scintillans* pop1‐pop3. Four newly sequenced *Paramecium* sequences (*P. primaurelia*, *P. sexaurelia*, and *P. caudatum* pop1‐2) group with previously sequenced ones.

### 3.2 Phylogenetic Analyses Inferred From SSU‐rRNA Gene, ITS1‐5.8S‐ITS2 Region, and LSU‐rRNA Gene Sequences (Figures 3, 4, 5, 6, 7)

Compared to concatenated trees (Figures 1 and 2), SSU‐rRNA gene tree shows similar topologies. However, phylogenetic assignments of some taxa are rather different. In the SSU‐rRNA gene tree (Figures 3 and 4), the subclass Hymenostomatia is not closely related to “SAA” but forms a sister to the Peritrichia (41% ML, 0.57 BI), whereas in the concatenated tree (Figures 1 and 2) it is closely related to “SAA”. *Cinetochilides ovale* (Loxophalidae) and *Pseudocollinia beringensis* (Apostomatia) (100% ML, 1.00 BI) form sister clades to the subclass Scuticociliatia (78% ML, 1.00 BI) (Figure 4), different to their position as sisters to Hymenostomatia‐“SAA” in the concatenated tree (Figures 1 and 2). Within subclass Peritrichia, order Mobilida forms a sister clade with the Peniculia instead of order Sessilida (Figure 4). The subclass Urocentria, represented by *Urocentrum turbo* , is sister to Hymenostomatia‐Peritrichia (42% ML, 0.62 BI) (Figures 3 and 4), whereas it is a sister to Hymenostomatia‐SAA in the concatenated tree (82% ML, 1.00 BI) (Figures 1 and 2). The placements of all newly sequenced sequences in the SSU‐rRNA gene tree resemble those in the concatenated tree.

Compared to the concatenated tree (Figures 1 and 2), the ITS1‐5.8S‐ITS2 rRNA tree (Figures 5 and 6) reveals slight disparities in their topologies. The primary topological differences are observed within the Scuticociliatia. Notably, *I. multifiliis* falls into the genus *Tetrahymena* rather than forming a sister clade with it. Similar to the concatenated tree, the subclass Peritrichia and Peniculia are sister taxa; however, instead of forming a new sister clade with Hymenostomatia‐“SAA,” Peritrichia and Peniculia are grouped solely as sister taxa with Hymenostomatia. Additionally, the subclasses Urocentria and Trichodinidae form a sister clade (88% ML, 0.93 BI) situated at the base of Peritrichia. The subclass Apostomatia, represented by *Pseudocollinia beringensis*, is located within the order Philasterida. Additionally, for the first time, the subclass Astomatia separates from Scuticociliatia and is situated at the basal clade of the entire class Oligohymenophorea. The subclass Scuticociliatia is distinguished from Hymenostomatia as a non‐monophyletic group. The focus within subclass Scuticociliatia centers around the fragmented and dispersed order Loxcephalida within the ITS1‐5.8S‐ITS2 tree (Figure 6). This includes examples such as *Cinetochilides ovale* (Loxcephalida) positioned in the order Pleuronematida and the segregation of *Pseudoplatynematum denticulatum* and *Sathrophilus holtae* within the Pleuronematida and Philasterida, respectively. Furthermore, the sister clade *Cardiostomatella vermiformis* and *Paratetrahymena wassi* form a new sister clade with other species of Scuticociliatia.

Owing to the limited availability of sequences, the LSU‐rRNA gene tree exhibits a reduced number of sequences compared to the ITS1‐5.8S‐ITS2 and SSU‐rRNA gene trees. Nevertheless, their topologies exhibit slight deviations (see Figure 7). In the LSU‐rRNA gene tree, Urocentria shows a sister relationship with Hymenostomatia (79% ML, 1.00 BI). The scuticociliate order Loxcephalida divides into three clades: (i) *Cardiostomatella vermiformis* + *Paratetrahymena wassi* as a sister clade to scuticociliates, excluding *Cinetochilides ovale*; (ii) *Pseudoplatynematum denticulatum* + *Sathrophilus holtae* as a sister clade to the orders Pleuronematida + Philasterida; (iii) *C. ovale* clusters with *Pseudocollinia beringensis* (99% ML, 1.00 BI). *C. ovale* + *P. beringensis* forms a sister to the Peritrichia (53% ML, 1.00 BI), whereas in the concatenated tree (Figures 1 and 2) it is closely related to Hymenostomatia and Scuticociliatia. They subsequently form a new sister clade with Hymenostomatia and Scuticociliatia (see Figure 7).

### 3.3 Phylogenetic Analyses Inferred From mtSSU‐rRNA and Cox 1 Gene Sequences (Figures 8, 9, 10; Figures S1 and S2)

The mtSSU‐rRNA gene tree comprises only sequences of subclasses Hymenostomatia, Scuticociliatia, and Peniculia. It consists of four lineages: (i) subclass Hymenostomatia; (ii) subclass Peniculia, encompassing *Protocyclidium sinica*; (iii) the scuticociliate order Philasterida, as well as several Pleuronematida species; (vi) some species in order Pleuronematida (Figure 8).

In the *cox1* gene tree, including sequences of four subclasses (Hymenostomatia, Scuticociliatia, Urocentria, and Peniculia). The relationship between Scuticociliatia and Peniculia is enhanced compared to the mtSSU‐rRNA gene tree, although with a limited number of sequences. Within subclass Hymenostomatia, the family Glaucomidae falls into the basal position, and the family Tetrahymenidae is divided into 11 groups (Figure 9). The sister clade relationship between Glaucomidae and Tetrahymenidae is also confirmed in the *cox 1* amino‐acid tree (Figure S1). Peniculia and Hymenostomatia form a sister clade, and Peniculia appears to be monophyletic (Figure 10). The subclass Urocentria emerges as a sister clade to Scuticociliatia (excluding *Cinrtochilum ovale*) (Figure 10). In the *cox1* amino‐acid tree, subclass Urocentria emerges as a sister clade to Hymenostomatia + Scuticociliatia (Figure S2).

Diverging from the mtSSU‐rRNA gene tree (depicted in Figure 8), the scuticociliate order Loxcephalida exhibits four available sequences, delineating into three distinct clades (Figure 10; Figure S2). These encompass *Dexiotricha* sp. and *D. elliptica*, forming a sister clade (99% ML, 1.00 BI) at the base of the order Philasterida; *Cardiostomatella vermiformis* groups as a sister clade to Philasterida and Pleuronematida; and *Cinetochilides ovale*, exclusively found in the context of Scuticociliatia's exterior (Figure 10). Similar topologies are exhibited in the *cox1* amino‐acid tree. As exceptions, *Dexiotricha* sp. + *D. elliptica* exhibit a slight positional variation, i.e., *Dexiotricha* sp. + *D. elliptica* (91% ML, 1.00 BI) as a sister to the scuticociliate order Philasterida as well as several Pleuronematida species (Figure S2).

### 3.4 ITS2 Secondary Structures

**FIGURE 11:**
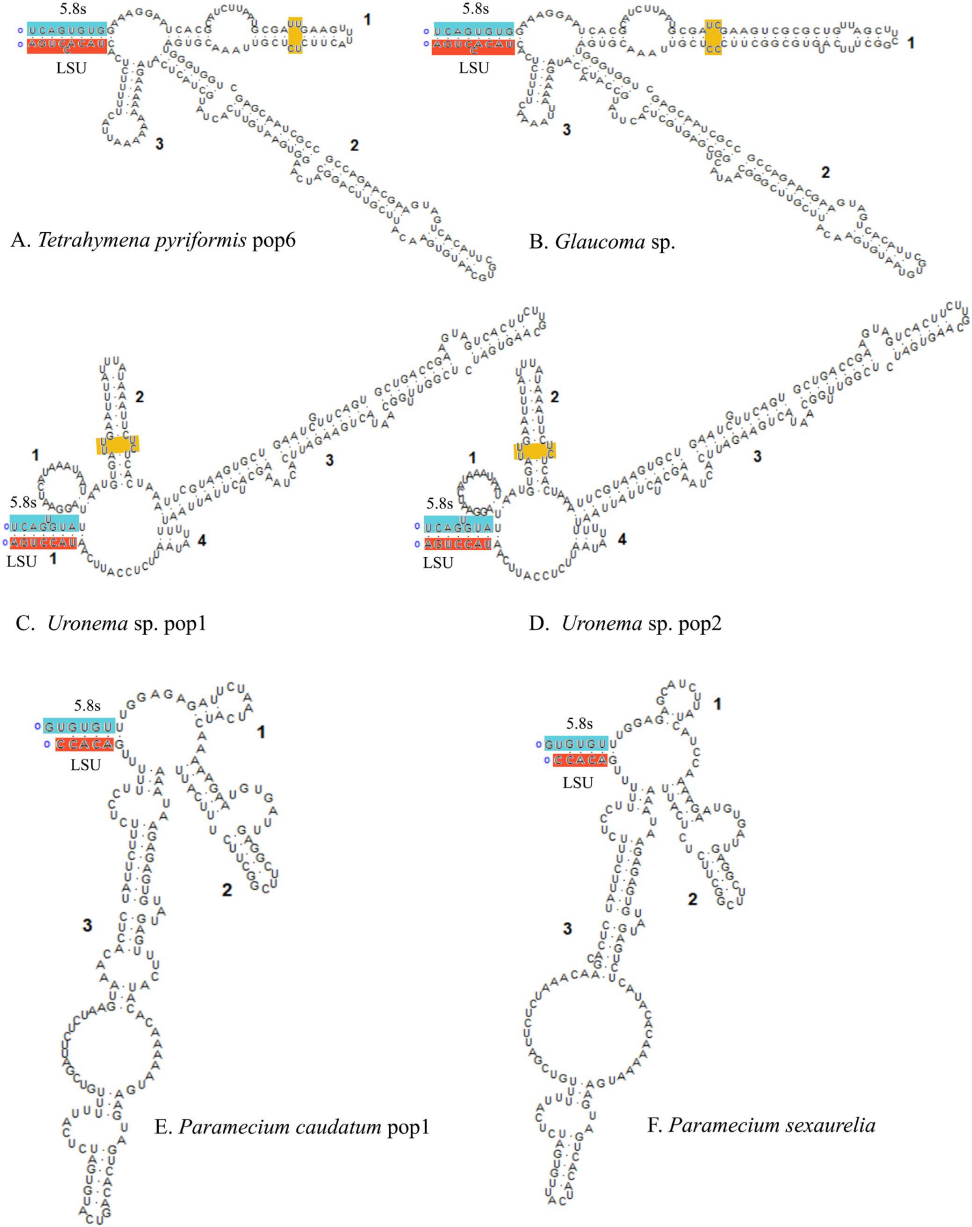
The putative ITS2 secondary structures of six oligohymenophorean species. The 5.8S sequences are in blue and the LSU‐rRNA gene sequence are marked in red. The helix's pyrimidine–pyrimidine mismatches are indicated by an orange rectangle.

The ITS2 secondary structures offer more comprehensive information than the sequence alone. Hence, ITS2 secondary structures of the six species, viz. *T. pyriformis* , *Glaucoma* sp., *Uronema* sp. pop1‐2, *Paramecium caudatum* , and *Paramecium sexaurelia*, are illustrated in Figure 11, displaying a similar pattern among the subclass. In Hymenostomatia, both *Tetrahymena* and *Glaucoma* are characterized by a large loop separated by three helices. Although the genus *Paramecium* also contains 3 helices, the longest helix in *Tetrahymena* and *Glaucoma* is helix 2, whereas that in *Paramecium* is helix 3. Structural variations in the genera *Tetrahymena* and *Glaucoma* are predominantly in helices 1 and 3. The model of *Paramecium* is: (i) helices 1–3 are both not conserved, and (ii) helix 2 and helix 3, although next to each other, are not fixed in position, and the unpaired spacer nucleotides between helices 1 and 2 are different (5′‐AA‐3′ vs. 5′‐AUCCA‐3′). *Uronema* is composed of a large loop separated by four helices. The genus *Uronema* exhibited four helices. Pyrimidine–pyrimidine mismatches are identified in helices 1 and 2 of Hymenostomatia and Scuticociliatia.

## 4 Discussion

### 4.1 Re‐Clarifying Phylogenetic Relationships Within Class Oligohymenophorea With New Sequences

As revealed in previous investigations (REF), the present study has revealed that individual gene trees exhibit diverse and ambiguous clustering patterns. For instance, the SSU‐rRNA gene tree illustrates that two peritrichous *Trichodina* species fall into the subclass Peniculia (Figure 4, Triangle hint), while the ITS1‐5.8S‐ITS2 tree showcases *Lembadion* sp. (subclass Peniculia) within the Peritrichia (Figure 6, Square hint). In the concatenated tree, monophyly of subclasess is revealed (Figures 1 and 2). Additionally, single‐gene trees may exhibit various clustering patterns among different studies, while the multiple gene trees show nearly congruent topologies (e.g., Gao, Katz, and Song 2012; Rataj, Zhang, and Vd'ačný 2022; Zhang et al. 2019). All these validate values of employing multigene trees to elucidate relationships within Oligohymenophorea. In the present investigation, more diverse species were sampled to improve the topologies of multigene trees.

Here, a total of 97 new sequences covering three oligohymenophorean subclasses (Hymenostomatia, Scuticociliatia, and Peniculia) are obtained, and multigene trees including three nuclear genes and two mitochondrial genes are reconstructed. Our phylogenetic topologies are similar to previous multigene trees. Within Hymenostomatia, Glaucomidae is nested within Tetrahymenidae, thereby rendering Tetrahymenidae non‐monophyletic (Rataj and Vďačný 2020; Zhang and Vd'ačný 2022). After the addition of newly sequenced *Tetrahymena glochidiophila* and *T*. *unionis* (Harbin populations), the paravorax group in *Tetrahymena* does not reintegrate into the borealis and australis groups. The genus *Tetrahymena* remains non‐monophyletic. Within subclass Scuticociliatia, the clustering patterns of four newly sequenced *Uronema* species further confirm the paraphyly of genus *Uronema* (Gao et al. 2016; Pan et al. 2020; Zhang et al. 2019; Hao et al. 2022). Two Harbin populations of *Pseudocohnilembus persalinus* collected from freshwater habitats mix with previously sequenced populations from brackish environments. This indicates that habitat type is not a principal factor in differentiating between species (Song 2000; Pan et al. 2015; Zhang et al. 2019). For *Paramecium* species, the multigene trees (Figure 2, five‐pointed star hint) indicate that *P. caudatum* has a close relationship with *P. sexaurelia*. However, the relationships of *P. caudatum* with *P. jenningsi* and *P. aurelia* require further evidence for clarification.

### 4.2 The Main Clades Within Class Oligohymenophorea

**FIGURE 12:**
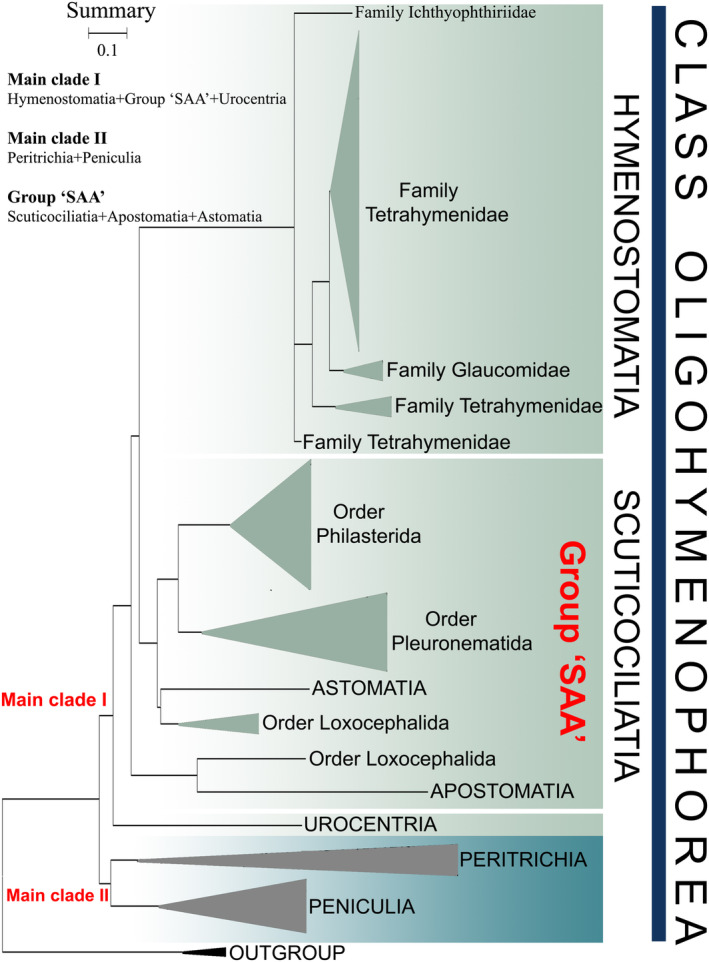
The summary figure of phylogenetic relationships within the class Oligohymenophorea (as depicted in Figures 1 and 2) illustrating major taxonomic groups at or above the family level. It delineates two principal branches and the group‘SAA’.

Currently, the class Oligohymenophorea predominantly comprises two main clades (Figure 12): (I) clade I, including subclasses Hymenostomatia, Urocentria, and Scuticociliatia, Apostomatia, and Astomatia (SAA); and (II) clade II, containing subclasses Peniculia and Peritrichia. Clade I is also supported by similar morphological characters, such as somatic kinetids (Small and Lynn 1981, 1985) and particle array patterns within the ciliary membranes (Bardele 1981). Notably, no sequence is available for the subclass Astomatia and Apostomatia in either our mtSSU‐rRNA (Figure 8) or previous SSU‐rRNA gene trees (Feng et al. 2015). However, our analyses show that including them in the concatenated tree does not disrupt the arrangement, and Urocentria is found to be in the basal position within this clade (Figure 2). As shown in previous studies (Feng et al. 2015; Jiang et al. 2019, 2024), our concatenated trees (Figures 1 and 2) reveal that clade II containg Peritrichia and Peniculia clusters at the base of the class Oligohymenophorea. Morphologically, Peritrichia and Peniculia share a similar oral region structure, the paroral and the three adoral polykinetids. Simultaneously, the depressed oral region distinguishes them from Hymenostomatia and Scuticociliatia. For instance, the Tetrahymenidae within Hymenostomatia, as well as the Philasteridae and Uronematidae within Scuticociliatia, present shallow depressions in their oral regions (Lynn 2008).

The subclass Hymenostomatia is regarded as monophyletic and stable within the terminal clade of Oligohymenophorea. However, its sister clade varies depending on different phylogenetic trees. In the mtSSU‐rRNA gene tree, Hymenostomatia is sister to the Scuticociliatia and shows close relation to the Peritrichia in the SSU‐rRNA gene, the Peniculia in the *cox 1* gene, the Urocentria in the LSU‐rRNA gene, and Peritrichia‐Peniculia in ITS1‐5.8S‐ITS2. Hymenostomatia and Peritrichia cluster together in the SSU‐rRNA gene tree, which is consistent with the previous studies (Feng et al. 2015; Miao et al. 2004). However, in the phylogenomic trees conducted by Feng et al. (2015) and Wang et al. (2021), and the concatenated trees by Rataj, Zhang, and Vd'ačný (2022), Zhang and Vďačný (2024), and Zhang et al. (2019), Hymenostomatia and Scuticociliatia cluster together, branching pattern of which aligns with those in our concatenated and mtSSU‐rRNA gene trees. In addition, the phylogenomic tree presented by Jiang et al. (2024) illustrates that Hymenostomatia first clusters with Urocentria, subsequently evolving into a distinct sister clade alongside Scuticociliatia. Morphologically, the subclasses, Hymenostomatia and Scuticociliatia exhibit commonalities in their oral apparatus, including a dikinetid paroral and three oral polykinetids (Lynn 2008). Additionally, they share consistent patterns of somatic kinetids (Small and Lynn 1981, 1985) and similar particle array patterns within the ciliary membranes (Bardele 1981). Based on the combination of morphological information and multigene analyses, we infer that the Scuticociliatia form a sister clade to Hymenostomatia, supporting the claim that multigenes can accurately reflect ciliate phylogeny compared to individual genes.

The ‘SAA’ group includes the subclasses Scuticociliatia, Astomatia, and Apostomatia. Morphologically, Astomatia is characterized by its mouthless form, while Apostomatia exhibits highly modified oral structures, setting them apart from other oligohymenophorean species. Lynn (2008) proposed that evolutionary divergence is the primary factor responsible for the observed morphological and molecular disunity between Scuticociliatia, Astomatia, and Apostomatia. Scuticociliatia, Astomatia, and Apostomatia are closely related; many of their species share cylindrical body shapes and symbiotic life histories (Lynn 2008). The positions of Astomatia and Apostomatia are gradually becoming clearer as more sequences are incorporated. Nevertheless, only a limited number of sequences are available for subclass Astomatia and Apostomatia thus far, and the broad sampling and sequencing are necessary to reveal a more comprehensive genealogical relationship.

### 4.3 Phylogeny of Tetrahymenidae

Initially, *Tetrahymena* were classified into three groups: the *pyriformis*, the *rostrata*, and the *patula* complexes. However, *Tetrahymena* species in the phylogenetic tree do not distinctly group into three clades. Previous studies suggest a classification of the *Tetrahymena* genus into two primary lineages (the borealis group and australis group) (Corliss 1970; Doerder 2019; Lynn et al. 2018; Pitsch et al. 2017; Rataj and Vďačný 2020), along with a paravorax group with fewer species based on SSU‐rRNA genes (Doerder 2019; Liu et al. 2023; Strüder‐Kypke et al. 2001; Zhang and Vďačný 2021).

The positioning of *Tetrahymena rostra* within these groups appears unstable with varying assertions from different studies, placing it (1) within the borealis group (Rataj and Vďačný 2020; Zhang and Vd'ačný 2022), (2) inside the australis group (Liu et al. 2023), or (3) outside the traditional groups (Watt et al. 2021). In our investigation, the accurate placement of *T. rostra* is challenging in the single‐gene trees (Figures 3, 5, and 7, Triangle hint). And in the multigene tree (Figure 1, Triangle hint), *T. rostra* aligns with the australis group and clusters with *T. caudata* , corroborating the findings of Liu et al. (2023). Also, Figure 8 of Rataj and Vďačný (2020), which indicates a slight trend toward a parasitic lifestyle in *T. caudata* and a substantial lifestyle shift in *T. rostra*, further supports *T. rostra* association with the australis group. For newly sequenced species, the multigene trees show more reasonable topologies compared to other single‐gene trees, which is most evident in the location of *T. setosa* and *T. pyriformis* . Four *T. setoa* populations clustered together in the multigene tree (Figure 1, Red line hint). By contrast, *T. setosa* pop4 is separated from *T. setosa* pop1‐3 in SSU‐rRNA gene trees (Figure 3, Double triangle hint), and it nests within *T. pyriformis* in LSU‐rRNA and mtSSU‐rRNA gene trees (Figures 7 and 8, Double triangle hint). However, the occurrence of *T. tropicalis* pop6 in the *T. pyriformis* species is potentially erroneous due to insufficient sampling of the relevant sequences (Figure 1, Question mark hint). These sequences encompass only ITS1‐5.8S‐ITS2 and *cox 1* genes, which may not be sufficient for accurate classification. Presently, Tetrahymenidae seems to be non‐monophyletic, because Glaucomidae consistently nests between the paravorax group and the borealis, australis groups. The position of the paravorax group remains controversial, probably due to sampling imbalances. A more comprehensive investigation, including diverse species within the paravorax group, is necessary to ascertain the optimal position for this group.

### 4.4 Topics of Controversy: Phylogenetic Positions of Subclass Urocentria and Order Loxocephalida

The subclass Urocentria was classified as an order within the Peniculia based on its ophryobuccokinetal stomatogenetic and oral polykinetid (Gao et al. 2016; Sun et al. 2021; Didier and Puytorac 1969; Lynn 1981). Wang et al. (2021) established Urocentria based on morphological and molecular data, alongside orthogonal analyses and a review of previous single‐ and multigene studies. It's a pity that Wang et al. (2021) did not include sequences of subclasses Apostomatia and Astomatia in their research. In the present study, the inclusion of subclasses Apostomatia and Astomatia revealed different patterns compared to previous investigations (whether derived from single or multiple genes). For instance, the multigene tree illustrates that Astomatia species fall into the order Loxocephalida (Gao, Katz, and Song 2013) or are grouped with the order Pleuronematida in the single‐gene tree (Poláková, Bourland, and Čepička 2023). However, in the present study, Astomatia was found to be located at the base of Loxocephalida (Figure 2). Morphologically, the subclass Urocentria is distinguished from the subclass Peniculia by its unique somatic monokinetids and the absence of nematodesmata (Corliss 1979). Despite these distinctions, similarities in stomatogenesis exist between Urocentria and Peniculia, along with the presence of microtubules (Didier 1970; Lynn 2008; Sundararaman and Hanson 1976). Given its interstitial morphological characteristics within Urocentria and its intermediary position in the concatenated tree (between Group II and the subclasses Hymenostomatia + cluster ‘SAA’), we regard it as a transitional taxon in the evolutionary path of the class Oligohymenophorea. Urocentria is the most primitive Group I and can be traced back to the subclass Peniculia in terms of its morphology.

The second topic of controversy is whether Loxocephalida is monophyletic (Gao, Katz, and Song 2013; Gao et al. 2016; Poláková, Čepička, and Bourland 2021; Yi et al. 2009; Zhang et al. 2010, 2019). Family Cinetochilididae consistently appears separate from Loxocephalida and the entire Scuticociliatia subclass (Gao, Katz, and Song 2013; Poláková, Čepička, and Bourland 2021; Poláková, Bourland, and Čepička 2023 and the present study Figures 2, 4, and 7). The family Cinetochilididae was defined by Poláková, Čepička, and Bourland (2021), whose study also revealed a well‐supported sister relationship between *C. ovale* and Apostomatia in the concatenated tree. Morphologically, apart from the presence of a ribbed wall, Cinetochilididae seems to bear no further resemblance to Loxocephalida (either in terms of oral polykinetids or oral area) (Lynn 2008). This also explains why Cinetochilididae is always separated from Loxocephalida. We assume that loxocephalidas emerged as the earliest scuticociliatia species, followed by Pleuronematida and Philasterida. This assertion finds further support in morphological evidence, particularly in the positioning of the genus *Dexiotricha* at the base of Loxocephalida. Morphologically, akin to most species within Scuticociliatia, the parental paroral membrane in *Dexiotricha* undergoes the stomatogenetic process, undergoing division at an early stage, leading to the formation of the oral primordia (Song et al. 2005). Song et al. (2005) proposed placing this taxon between Scuticociliatia and Hymenostomatia. However, both in historical context and our current study, the sequences of this taxon are consistently found at the base of the subclass Scuticociliatia (Bourland et al. 2024; Gao, Katz, and Song 2013; Gao et al. 2016; Li et al. 2006). Integrating morphological information and multigene analyses, we deduce that the order Loxocephalida represents an early member of Scuticociliatia, potentially serving as a prototype for this taxonomic group.

### 4.5 Application of Evolutionary Taxonomy Concepts in Other Areas

Clarifying the relationships among species has the potential to significantly enhance our comprehension of biodiversity. For example, reliable interspecific relationships may enhance the stability of the phylogenetic tree, consequently improving the revision of biological classification systems (Corliss 1979; Lynn 2008). Also, clear phylogenetic relationships may have profound implications for ecological studies, considering that species interactions and niches are often intricately linked to their genetic relationships (Sun et al. 2021). Clarifying the evolutionary history of oligohymenophorean ciliates plays an important role in preventing diseases in aquaculture, as well as in the fields of parasitology and pathology (Raman and Park 2024). In the field of comparative genomics, new phylogenetic insights can provide clues about the evolutionary history of the species. By comparing the genomes of different species, it is possible to track the evolutionary paths of specific genes and understand how they spread and varied among different species (Jin et al. 2023; Zhang et al. 2014; Su et al. 2023, 2024). This will not only reveal the mechanisms by which species adapt to environmental changes, such as the transition of ciliates from free‐living to parasitic life or from marine to freshwater, but also provide a scientific basis for biodiversity conservation (Lian et al. 2023; Liu et al. 2023; Rataj and Vďačný 2020; Vd'ačný and Foissner 2019). Understanding the genetic diversity of species can help identify conservation priorities and ensure the conservation of key genes (Aapo, Mikael, and Janne 2014).

The limitations of sample collection have consistently posed a challenge within the realm of phylogenetic research, and unbalanced sampling can notably impact the results of phylogenetic analysis, potentially leading to para or polyphyletic topologies (Liu et al. 2023; Vd'ačný and Foissner 2019). For example, the family Tetrahymenidae remains non‐monophyletic due to the paravorax group being distinct from the borealis and australis groups (Doerder 2019; Rataj and Vďačný 2020; Strüder‐Kypke et al. 2001; Zhang and Vďačný 2021). The limited data available for the paravorax group impedes its integration into the borealis and australis branches. Therefore, these potential deviations and limitations must be considered when discussing study findings in order to ensure the accuracy and reliability of conclusions.

## Author Contributions

**Bailin Li:** formal analysis (lead), software (lead), writing – original draft (lead), writing – review and editing (equal). **Yumeng Song:** formal analysis (equal), investigation (equal), software (equal). **Xiang Wang:** formal analysis (equal), investigation (supporting). **Qiyue Zhao:** formal analysis (equal), investigation (supporting). **Menghan Liu:** formal analysis (supporting), investigation (equal). **Lihui Liu:** formal analysis (supporting), software (equal). **Xuming Pan:** conceptualization (equal), funding acquisition (lead), methodology (lead), supervision (lead), writing – review and editing (equal). **Zhenzhen Yi:** conceptualization (equal), methodology (equal), writing – review and editing (equal).

## Conflicts of Interest

The authors declare no conflicts of interest.

## Supporting information


**Figure S1.** Phylogenetic tree of subclass Hymenostomatia based on the cox 1 amino acid sequences. Owing to the large size of the image, the phylogenetic tree has been segmented into two parts, labeled as ‘Hymenostomatia’ and ‘Scuticociliatia, Astomatia, Apostomatia, Urocentria, Peniculia, Peritrichia’. A comprehensive view of the tree is presented in the lower left of the image, while the black section represents a portion of the original image (subclass Hymenostomatia). Newly sequenced species in this study are in red. The supports for nodes are indicated as follows: ML bootstraps/BI posterior probability. ‘‐’ indicates a mismatch in topology between Bayesian and ML trees. Fully supported (100%/1.00) clades are marked with solid circles. ‘*’ at nodes indicates the support values < 50%/0.5 (ML/BI). The scale bar corresponds to 0.5 expected substitutions per site.

**Figure S2.** Phylogenetic tree of subclass Scuticociliatia, Astomatia, Apostomatia, Urocentria, Peniculia, and Peritrichia based on the cox 1 amino acid sequences. Owing to the large size of the image, the phylogenetic tree has been segmented into two parts, labeled as ‘Hymenostomatia’ and ‘Scuticociliatia, Astomatia, Apostomatia, Urocentria, Peniculia, Peritrichia’. A comprehensive view of the tree is presented in the lower left of the image, while the black section represents a portion of the original image (subclass Scuticociliatia, Astomatia, Apostomatia, Urocentria, Peniculia). Newly sequenced species in this study are in red. The supports for nodes are indicated as follows: ML bootstraps/BI posterior probability. ‘‐’ indicates a mismatch in topology between Bayesian and ML trees. Fully supported (100%/1.00) clades are marked with solid circles. ‘*’ at nodes indicates the support values < 50%/0.5 (ML/BI). The scale bar corresponds to 0.5 expected substitutions per site.

## Data Availability

The data presented in the study are deposited in the NCBI database (https://www.ncbi.nlm.nih.gov/) repository; accession numbers, lengths, and G&C contents are shown in Tables 2 and 3. It may not be traceable until the date of release.
