# Supplementary figures and images for: A Reassessment of Phylogenetic Relationships in Class Oligohymenophorea (Protista, Ciliophora) Based on Updated Multigene Data
Source: Ecol Evol. 2025 Feb 24;15(2):e70950. doi: 10.1002/ece3.70950 (PMC11850450; doi:10.1002/ece3.70950)

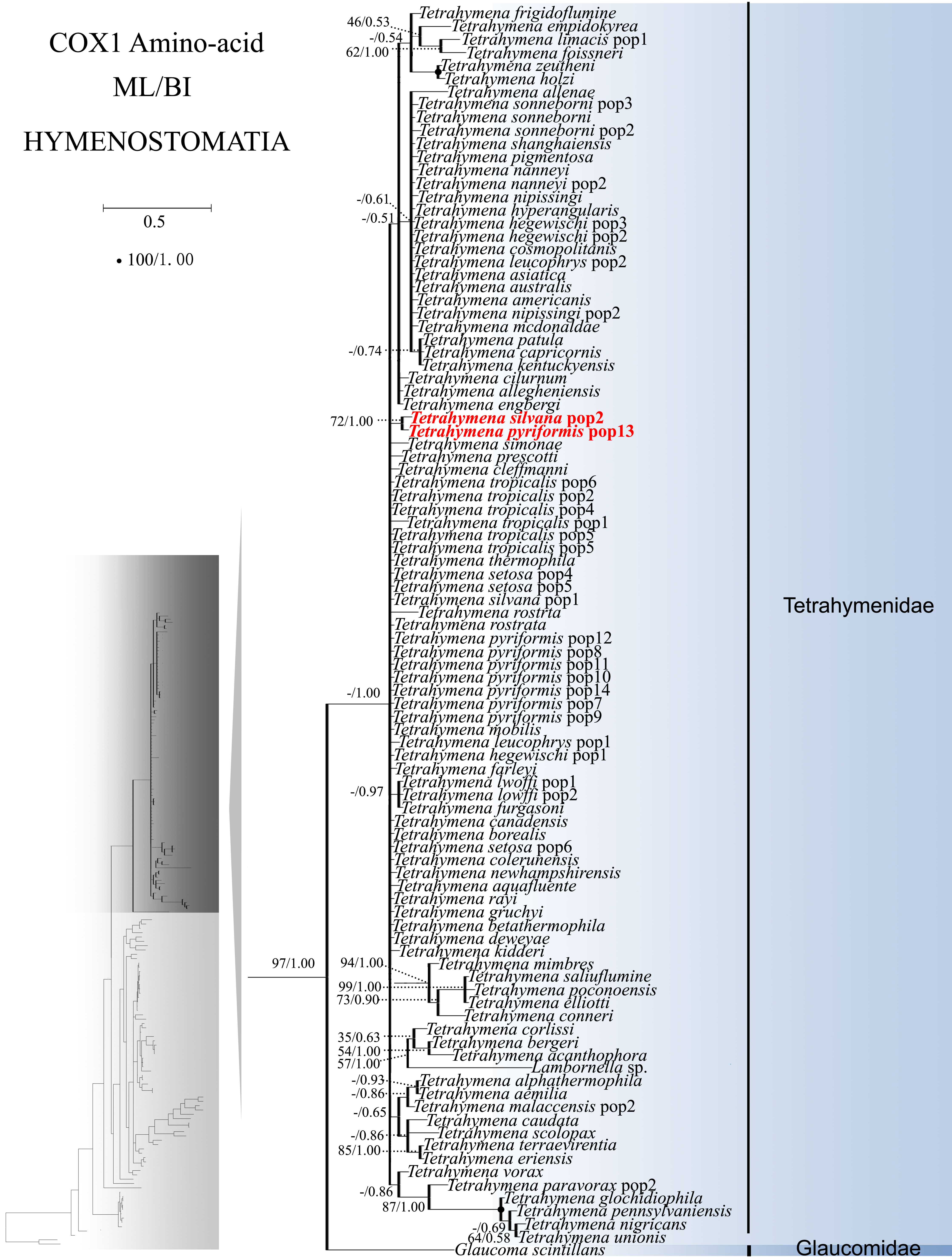

Supplement: Supplementary file 1 — Figure S1. Phylogenetic tree of subclass Hymenostomatia based on the cox 1 amino acid sequences. Owing to the large size of the image, the phylogenetic tree has been segmented into two parts, labeled as ‘Hymenostomatia’ and ‘Scuticociliatia, Astomatia, Apostomatia, Urocentria, Peniculia, Peritrichia’. A comprehensive view of the tree is presented in the lower left of the image, while the black section represents a portion of the original image (subclass Hymenostomatia). Newly sequenced species in this study are in red. The supports for nodes are indicated as follows: ML bootstraps/BI posterior probability. ‘‐’ indicates a mismatch in topology between Bayesian and ML trees. Fully supported (100%/1.00) clades are marked with solid circles. ‘*’ at nodes indicates the support values < 50%/0.5 (ML/BI). The scale bar corresponds to 0.5 expected substitutions per site. [file ECE3-15-e70950-s002.jpg]

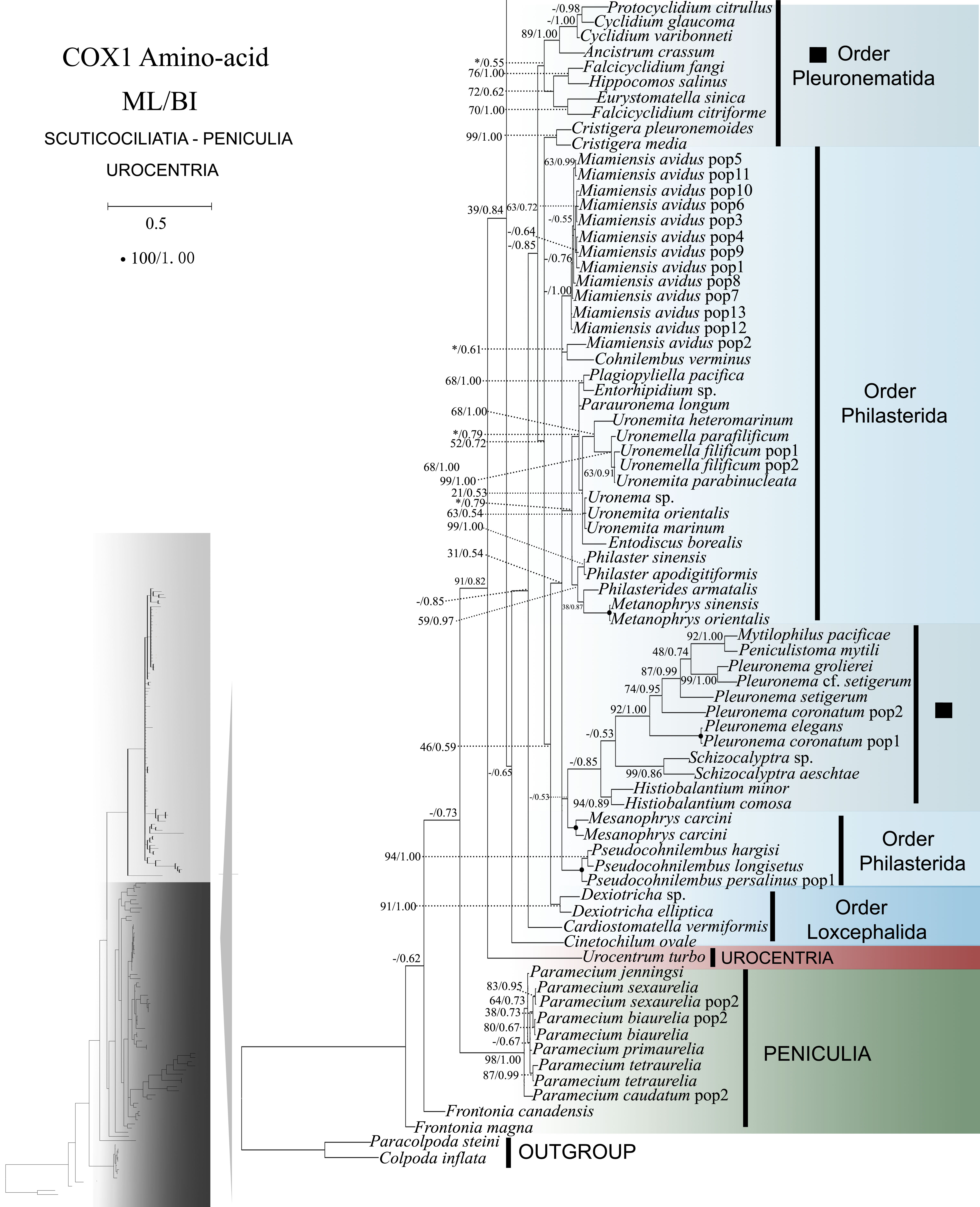

Supplement: Supplementary file 2 — Figure S2. Phylogenetic tree of subclass Scuticociliatia, Astomatia, Apostomatia, Urocentria, Peniculia, and Peritrichia based on the cox 1 amino acid sequences. Owing to the large size of the image, the phylogenetic tree has been segmented into two parts, labeled as ‘Hymenostomatia’ and ‘Scuticociliatia, Astomatia, Apostomatia, Urocentria, Peniculia, Peritrichia’. A comprehensive view of the tree is presented in the lower left of the image, while the black section represents a portion of the original image (subclass Scuticociliatia, Astomatia, Apostomatia, Urocentria, Peniculia). Newly sequenced species in this study are in red. The supports for nodes are indicated as follows: ML bootstraps/BI posterior probability. ‘‐’ indicates a mismatch in topology between Bayesian and ML trees. Fully supported (100%/1.00) clades are marked with solid circles. ‘*’ at nodes indicates the support values < 50%/0.5 (ML/BI). The scale bar corresponds to 0.5 expected substitutions per site. [file ECE3-15-e70950-s001.jpg]
